# Expression of oncogenic HRAS in human Rh28 and RMS-YM rhabdomyosarcoma cells leads to oncogene-induced senescence

**DOI:** 10.1038/s41598-021-95355-2

**Published:** 2021-08-13

**Authors:** Jenny J. Li, Alexander R. Kovach, Margaret DeMonia, Katherine K. Slemmons, Kristianne M. Oristian, Candy Chen, Corinne M. Linardic

**Affiliations:** 1 Duke University School of Medicine, Durham, NC USA; 2 Department of Pediatrics, Duke University School of Medicine, Durham, NC USA; 3 Department of Pharmacology and Cancer Biology, Duke University School of Medicine, Durham, NC USA; 4 Division of Pediatric Hematology-Oncology, Department of Pediatrics, Duke University School of Medicine, Box 102382 DUMC, Durham, NC 27710 USA

**Keywords:** Paediatric cancer, Sarcoma

## Abstract

Rhabdomyosarcoma (RMS) is the most common pediatric soft tissue sarcoma. The two predominant histologic variants of RMS, embryonal and alveolar rhabdomyosarcoma (eRMS and aRMS, respectively), carry very different prognoses. While eRMS is associated with an intermediate prognosis, the 5-year survival rate of aRMS is less than 30%. The RMS subtypes are also different at the molecular level—eRMS frequently has multiple genetic alterations, including mutations in *RAS* and *TP53*, whereas aRMS often has chromosomal translocations resulting in *PAX3-FOXO1* or *PAX7-FOXO1* fusions, but otherwise has a “quiet” genome. Interestingly, mutations in *RAS* are rarely found in aRMS. In this study, we explored the role of oncogenic RAS in aRMS. We found that while ectopic oncogenic HRAS expression was tolerated in the human RAS-driven eRMS cell line RD, it was detrimental to cell growth and proliferation in the human aRMS cell line Rh28. Growth inhibition was mediated by oncogene-induced senescence and associated with increased RB pathway activity and expression of the cyclin-dependent kinase inhibitors p16 and p21. Unexpectedly, the human eRMS cell line RMS-YM, a RAS wild-type eRMS cell line, also exhibited growth inhibition in response to oncogenic HRAS in a manner similar to aRMS Rh28 cells. This work suggests that oncogenic RAS is expressed in a context-dependent manner in RMS and may provide insight into the differential origins and therapeutic opportunities for RMS subtypes.

## Introduction

Rhabdomyosarcoma is a malignant tumor of mesenchymal origin characterized by skeletal muscle histogenesis. It is the most common soft tissue sarcoma of childhood, with about 350 cases per year in the United States^1^. The two predominant histologic variants of RMS, originally described because of their appearance under light microscopy, are embryonal (eRMS) and alveolar (aRMS). While eRMS is associated with an intermediate prognosis, aRMS is more aggressive and associated with a poorer prognosis^2,3^. Despite clinical trials, multi-disciplinary management, and improved supportive care, the 5-year survival rate for primary aRMS is as low as 33%, and the 5-year survival rate of recurrent or metastatic aRMS is < 10%^2,3^. This dismal prognosis has not improved in the last 40 years^2^.

We and others have sought to define the molecular abnormalities underlying RMS, with the goal that uniquely expressed proteins might be exploited as targets. While clinicians observed as early as the 1960s that eRMS and aRMS not only have different histologies, but often present at distinct anatomic sites and have differing responses to therapy, it has become clear that eRMS and aRMS also harbor distinct molecular defects. With the advent of next-gen sequencing, we now know that eRMS often has multiple genetic alterations, including mutations in *HRAS*, *KRAS*, *NRAS*, and *TP53*^4^. On the other hand, aRMS displays signature chromosomal translocations encoding *PAX3-FOXO1* or *PAX7-FOXO1* fusions^4^. Nomenclature is evolving and many groups are now using the terms “fusion-negative” to represent the eRMS family of tumors and “fusion-positive” to represent the aRMS family of tumors. Here, we use RAS-driven eRMS (R-eRMS) to represent classic eRMS, and PAX3-FOXO1-driven aRMS to represent classic aRMS. Since recent genomic landscape studies suggest that mutations in RAS or downstream RAS effectors are present in less than half of eRMS cases, while also identifying a subgroup of eRMS that have no apparent somatic mutations^5^, we also examined a RAS-wild type eRMS cell line for comparison.

RAS proteins are small GTPases that regulate a variety of cellular processes including cell cycle progression, cell survival, polarity, movement, and intracellular transport. RAS proteins carry out these functions by acting as molecular switches, alternating between the active GTP-bound and the inactive GDP-bound states. While their function is tightly regulated in normal cells, oncogenic *RAS* mutations yield constitutively active proteins that stimulate uncontrolled proliferation and survival in tumor cells^6^. Beginning in 1989, there were scattered reports of *RAS* mutations in human RMS specimens and cell lines^7–9^. The histologic type was not always stated, but in many cases *RAS* mutations were noted in samples with eRMS histology. In 2003, we described a role for *HRAS* in the genesis of eRMS using a genetically defined model based on transformation of human myoblasts^10^. In 2006, the genetic basis for Costello syndrome (characterized by developmental delay, elastic skin, and a propensity for tumors of embryonal origin including eRMS) was shown to be a constitutional oncogenic mutation in one *HRAS* allele^11^. Recent genomic analyses of RMS suggest that RAS pathway mutations may be associated with higher risk patients in eRMS, although the number of tumors studied was low^4,5^. It is notable that over the last three decades of studies, *RAS* mutations in aRMS tumors have rarely been found. This suggests that oncogenic RAS is either not required for tumorigenesis of aRMS or is not tolerated in aRMS.

In addition to its tumor-promoting role, RAS participates in oncogene-induced senescence (OIS). First described in 1997, OIS occurred when oncogenic RAS was expressed in rodent or human fibroblasts, leading to permanent growth arrest indistinguishable from replicative cellular senescence^12^. This growth arrest was associated with increases in the levels of the DNA-damage response sensor p53 and the cyclin dependent kinase inhibitors p21 and p16 and decreases in the levels of phospho-RB^12^. However, inactivation of either p16 or p53 abrogated RAS-induced senescence^12^. Taken together, OIS is a cellular defense mechanism that prevents tumorigenesis and requires intact RB/p53 pathways to be activated. Indeed, OIS is observed in many pre-malignant lesions but rarely observed in malignant tumors^13–18^. Of the three main RAS effector pathways (MAPK/ERK, PI3K/AKT, RALA/B), the MAPK/ERK and the PI3K/AKT pathways play crucial roles in OIS^14,19–21^.

Here, we investigated the role of RAS in Rh28 aRMS cells, since although RAS mutations are common in eRMS, they are rare in aRMS cells expressing PAX3-FOXO1. We reasoned that either oncogenic RAS is not required for tumorigenesis of aRMS or is not tolerated in aRMS. We found that ectopic expression of oncogenic HRAS is detrimental to Rh28 cell growth, leading to OIS. Using ectopic expression of activating mutants of RAS effectors, we found that activation of either the PI3K/AKT or MAPK/ERK pathway contributed to Rh28 cell growth inhibition. We then examined the RAS wild-type eRMS cell line, RMS-YM^22^, and unexpectedly found it responded in a similar fashion to Rh28 cells, with growth inhibition in response to both oncogenic HRAS or activated RAS effector mutants. Conversely, the R-eRMS RD cell line, which harbors an N-RAS mutation, was unaffected phenotypically by ectopic expression of oncogenic H-RAS. This differential tolerance of oncogenic RAS signaling suggests that oncogenic RAS is expressed in a context-dependent manner in RMS, a principle noted previously for RMS^23^, and may provide insight into the differential origins and therapeutic opportunities for RMS subtypes.

## Materials and methods

### Generation of cell lines and constructs

Human skeletal muscle myoblasts (HSMMs; Lonza) were cultured as described^24^. Human RMS cell lines RD^25^, Rh30^26^ and Rh28^27^ were gifts from Tim Triche (Children’s Hospital of Los Angeles, CA, USA) in 2005; Rh3^28^, Rh36^29^, and SMS-CTR^30^ were gifts from Brett Hall (Columbus Children’s Hospital, OH, USA) in 2006; RMS-YM RAS wild type cells were a gift from Marielle Yohe (Pediatric Oncology Branch, NCI, Bethesda, MD, USA); all human RMS cell lines were cultured as described^31^. Cell line authentication was performed in 2014 using STR analysis (Promega PowerPlex 18D) conducted by the DNA Analysis Facility at Duke University (Durham, NC, USA); Rh28 and Rh30 were reauthenticated in 2016. Of note, from the STR analysis, we retrospectively discovered that the Rh3 and Rh28 cell lines were derived from the same patient^32^ and this was originally described in Ref.^33^. For serum stimulation studies, cells were serum-starved for 24-48 h, followed by induction with 10% FBS for 1 h^36^; immunoblots were carried out to assess protein expression levels pre-and post serum stimulation.

H-RAS 12V with an N-terminal FLAG tag (oncogenic RAS)^34^, H-RAS-WT (wild type RAS), and H-RAS-17N (dominant negative RAS)^35^ expression plasmids with validated RAS activity were gifts from Chris Counter (Duke University Medical Center, NC, USA) and were subcloned into the pBabe-puro retroviral backbone. The RAS effector activating mutant plasmid encoding a myristoylated AKT, pBabe-puro-FLAG-MyrAKT (plasmid #15294), was purchased from Addgene. Other RAS effector activating mutant plasmids (pBabe-puro-HA-MEK1DD) were gifts from Chris Counter. Plasmids were verified by DNA sequencing. Retroviral particles were produced from HEK293T cells transiently transfected with the retroviral expression plasmid, pCL-10A1 (Imgenex), and Fugene 6 (Promega). Retroviral particles were harvested 48 h post transfection and filtered through 0.45 µM filters. Polybrene was added to viral particles to a final concentration of 4 µg/mL. Cells were stably infected with amphotrophic retroviruses derived from pBabe-puro, pBabe-puro-FLAG-H-RAS-12V, pBabe-puro-H-RAS-WT, and pBabe-puro-H-RAS-17N, pBabe-puro-FLAG-MyrAKT, and pBabe-puro-HA-MEK1DD. Rh28 cells were selected with 1 μg/mL puromycin (Sigma) for 2 days and RD cells were selected with 2 μg/mL puromycin for 2 days. RMS-YM cells were selected with 0.5 μg/mL puromycin for 2 days.

### Immunoblotting

Immunoblotting was performed as previously described, using a range of 30–100 µg of lysate per sample^36^. The following antibodies were used for immunoblotting: anti-pan-RAS (Calbiochem OP40, 1:1000), anti-pRB (Cell Signaling #8180, 1:1000), anti-RB (Cell Signaling #9313, 1:1000), anti-p16 (BD #554079 1:1000), anti-pp53 (Calbiochem PC461 1:5000), anti-p53 (Santa Cruz sc-126 1:200), anti-p21 (Santa Cruz sc-6246 1:500), anti-pAKT (Cell Signaling #9271 1:1000), anti-AKT (Cell Signaling #9272 1:1000), anti-pERK (Cell Signaling #9101 1:1000), anti-ERK (Santa Cruz sc-93 1:1000), and anti-actin (Sigma #A2066, 1:200). Total RB, pRB, total p53, pp53, p16, and p21 immunoblots were repeated three times each.

### Growth curve and BrdU assay

Cell growth was assayed using Trypan blue staining followed by manual cell counting on a hemocytometer. Cells were cultured in 6 cm dishes and counted at three or four time points between days 1 through 7 in triplicate. 1.5 × 10^5^ cells were plated per replicate at each time point. Cell proliferation was measured using BrdU assays as previously described^31^.

### Senescence and differentiation assays

Cell senescence was assessed by expression of β-galactosidase by light microscopy, as previously described^31^. In brief, cells were plated at equal density in 6-well plates and the following day were assayed for β-gal staining using an X-Gal-based senescence detection kit (Calbiochem) according to the supplier’s protocol. Myogenic differentiation was assessed by MF20 staining under light microscopy, as previously described^37^. In brief, cells were plated at equal density to about 60% confluence in 6-well plates and subject to differentiation-inducing conditions (fusion media consisting of DMEM/F12 with 2% horse serum and refreshed every other day for ∼ 5 days). Myotubes were fixed, permeabilized, and stained with primary antibody anti–sarcomere-myosin hybridoma MF20 followed by secondary biotinylated anti-mouse IgG, then HRP-streptavidin with a 3,3′-diaminobenzidine reagent. MF20 was obtained from the Developmental Studies Hybridoma Bank, created by the NICHD of the NIH and maintained at The University of Iowa, Department of Biology, Iowa City, IA 52242.

### RAS-GTP pulldown assays

**Figure 1:**
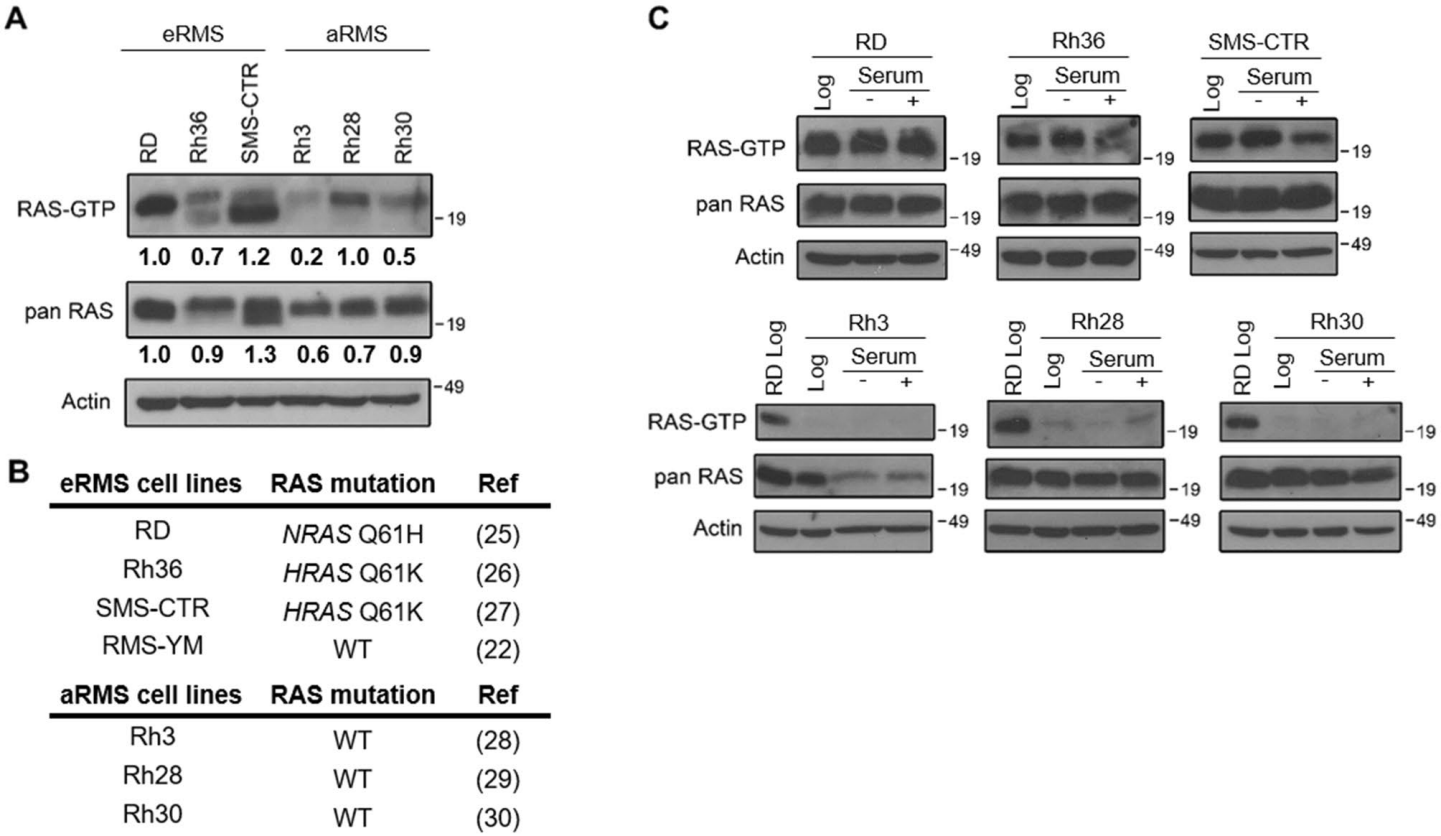
Activated RAS expression is lower in aRMS cells than eRMS cells. Expression of activated RAS and total RAS was examined by immunoblot in three R-eRMS (RAS-driven) cell lines (RD, Rh36, SMS-CTR) and three aRMS cell lines (Rh3, Rh28, Rh30). (**A**) Under log phase growth conditions, aRMS cell lines displayed lower levels of RAS-GTP than eRMS cell lines. Densitometry quantification is shown either for RAS-GTP/panRAS or panRAS and is normalized to the RD cell line. (**B**) Of the R-eRMS cell lines, RD cells harbor a *NRAS* mutation, and Rh36 and SMS-CTR cells harbor an *HRAS* mutation. The aRMS cell lines have wild type RAS. RMS-YM cells, examined later in the text, are wild type for *RAS*. (**C**) Under serum stimulation conditions, RAS-GTP levels were still lower in aRMS cell lines (bottom) than eRMS cell lines (top). Cells were collected either in log phase, after 24 h of serum starvation, or 24 h of serum starvation followed by 1 h of growth in serum media. Actin used as a loading control. RD cells collected in log phase were included in the aRMS blots (bottom) as a positive control.

The RAS-GTP pulldown assay in Fig. 1 was performed based on published methods^38,39^. Bacteria pellets expressing pGEX-RasBD (binding domain) were sonicated and the supernatant was incubated with prewashed glutathione sepharose-4B (GE) slurry for two hours at 4 °C. Beads were then washed with PBST buffer × 2 and resuspended in 1:1 ratio buffer to beads. 25 µL of GST-RasBD were incubated with 400 µg cell lysate for 45 min at 4 °C. After washing the lysate/bead mixture with lysis buffer, 30 µL of 2 × SDS-PAGE sample loading buffer was added to each sample. The samples were boiled for 8 min, followed by immunoblot. The RAS-GTP pulldown assay in Fig. 6A was performed using the Active Ras Detection Kit (Cell Signaling #8821).

### Statistics

Data is presented as the mean ± SD. Statistical analysis was performed using GraphPad Prism software (GraphPad); one-way ANOVA test was used. A P-value of less than 0.05 was considered significant.

## Results

### Activated RAS expression is lower in aRMS cells than R-eRMS cells

We first examined overall RAS protein expression in human R-eRMS (RD, Rh36, SMS-CTR) compared to human aRMS (Rh3, Rh28, Rh30) cell lines growing in log phase using immunoblot (Fig. 1A, Supplementary Fig. S1). Pan-RAS levels were lower in all three aRMS cells compared to all three R-eRMS cells, and densitometric quantitation revealed that activated RAS (RAS-GTP) was lower in two of the three aRMS cell lines. Since RD cells contain an *NRAS* mutation^32^, and Rh36 and SMS-CTR cells contain *HRAS* mutations^40,41^, higher levels of RAS-GTP are expected. On the other hand, none of the aRMS cell lines contain mutant RAS^32,42^ (Fig. 1B). RAS is crucial to cell cycle progression and the levels of activated RAS increase after serum-stimulated cell cycle entry^43^. To determine the extent to which RAS-GTP levels could be boosted in aRMS cells, we serum-starved all six cell lines for 24 h, followed by serum stimulation for one hour, and measured RAS-GTP levels pre- and post-stimulation (Fig. 1C, Supplementary Fig. S2). These conditions were chosen based on serum-stimulation of a control myogenic cell line, in which serum stimulation results in a robust RAS-GTP signal (Supplementary Fig. S3). For each cell line, the same cells growing in log phase were used as control. Once again, the baseline RAS-GTP levels were lower in aRMS cell lines compared to R-eRMS cell lines. Upon serum-stimulation, the R-eRMS cell lines were as predicted not able to generate additional RAS-GTP (since the endogenous oncogenic RAS was already increasing RAS-GTP to threshold). On the other hand, the aRMS cells were also minimally able to be stimulated, with only the Rh28 cells showing a mild increase in RAS-GTP expression. In sum, these results suggest that baseline RAS signaling activity is lower in aRMS cells compared to R-eRMS cells and that aRMS cells do not robustly activate RAS in response to serum-stimulation.

### Expression of oncogenic HRAS inhibits growth and proliferation in Rh28 aRMS cells

**Figure 2:**
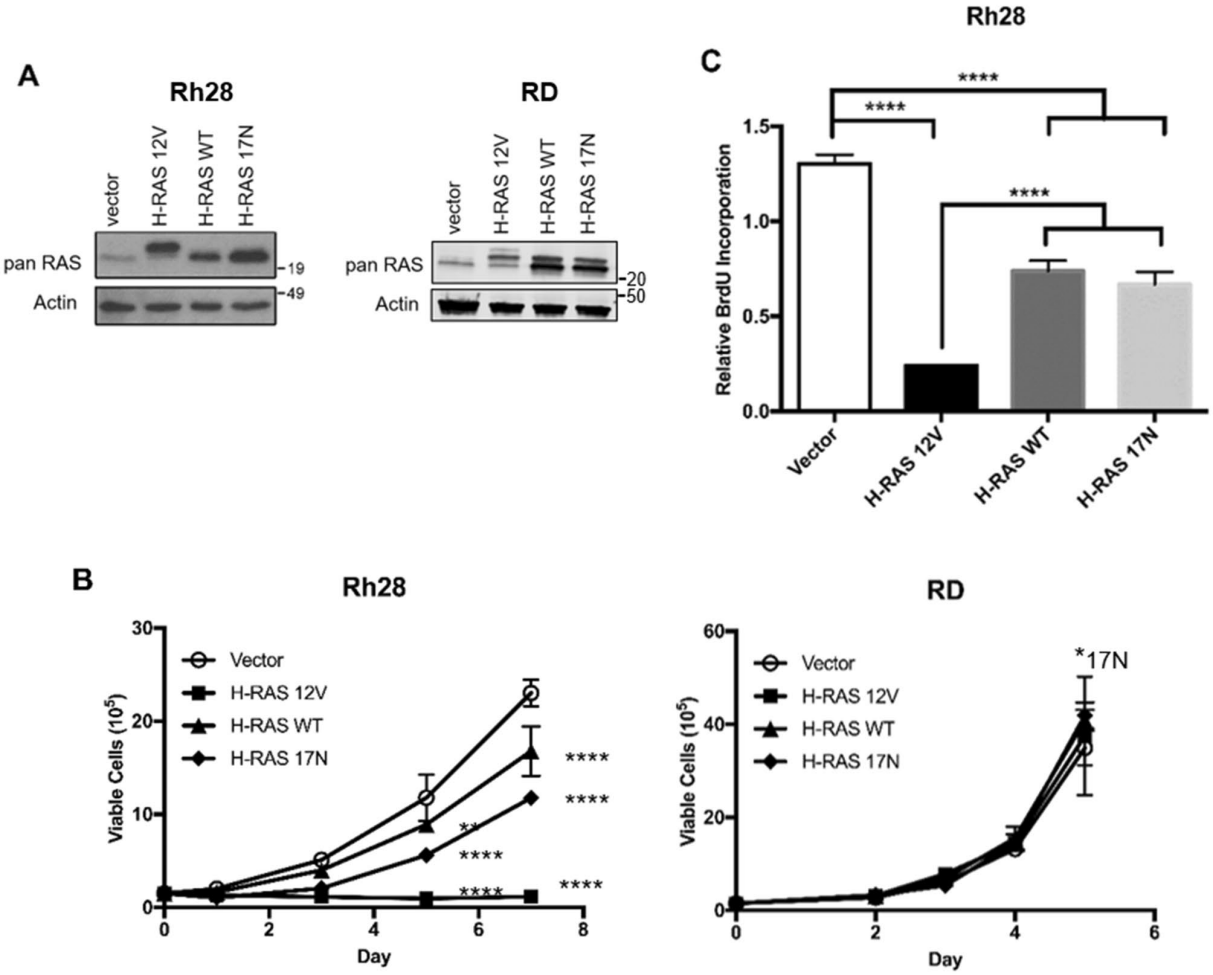
Stable expression of oncogenic H-RAS inhibits growth and decreases proliferation in Rh28 aRMS cells. (**A**) H**-**RAS mutants including oncogenic H-RAS (H-RAS 12V), wild type H-RAS (H-RAS WT), and dominant negative H-RAS (H-RAS 17N) were stably expressed in Rh28 aRMS and RD R-eRMS cells. RAS expression was measured by immunoblot using a pan-RAS antibody with actin as loading control. The H-RAS V12 band runs higher on SDS-PAGE because it contains an N-terminal FLAG tag. (**B**) Expression of oncogenic H-RAS caused growth suppression in Rh28 but not RD cells. Wild type and dominant negative H-RAS also impaired growth in Rh28 cells, though not to the same degree as oncogenic H-RAS. (**C**) Rh28 cell proliferation decreased in response to expression of each H-RAS mutant, especially oncogenic RAS, as measured by BrdU assay. (*p < 0.05; **p < 0.01; ****p < 0.0001).

To assess the phenotypic impact of altered RAS signaling in aRMS, we stably expressed wild type, dominant negative, or oncogenic *HRAS* mutants in Rh28 aRMS cells compared to RD R-eRMS cells. We chose *HRAS* since this was the isoform we had used to model eRMS from primary human myoblasts^10^. Expression of oncogenic HRAS (H-RAS 12V), wild type HRAS (H-RAS WT), or dominant negative HRAS (H-RAS 17N) were confirmed via immunoblot using a pan-RAS antibody (Fig. 2A, Supplementary Fig. S4) since isoform specific antibodies were not available, then cells expressing these constructs were examined for changes in growth. While both wild type and dominant negative HRAS moderately impaired the growth of Rh28 cells, expression of oncogenic HRAS caused growth arrest (Fig. 2B, left) and inhibition of proliferation as assessed by BrdU incorporation (Fig. 2C). On the other hand, growth of RD cells was unaffected regardless of the RAS mutant expressed (Fig. 2B, right).

### Expression of oncogenic HRAS increases differentiation in Rh28 aRMS cells

**Figure 3:**
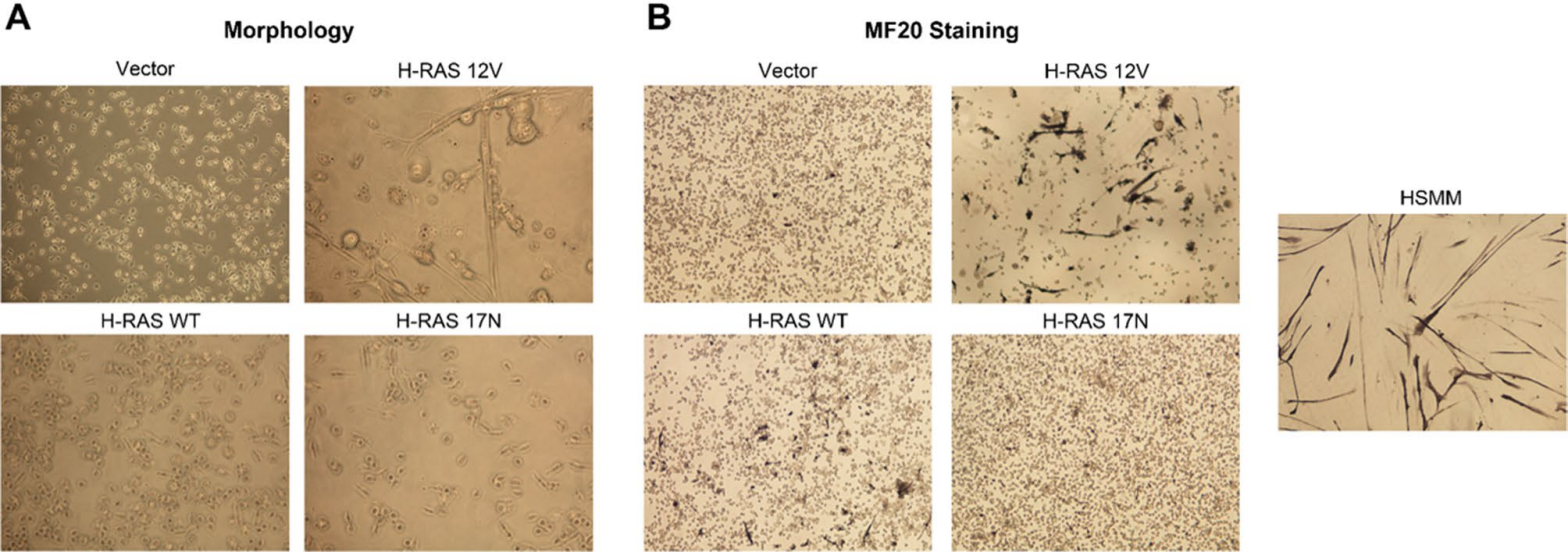
Expression of oncogenic H-RAS increases myogenic differentiation in Rh28 aRMS cells. (**A**) Under light microscopy, cells expressing oncogenic H-RAS 12V displayed an elongated morphology reminiscent of differentiating myotubes. (**B**) To assess for markers of differentiation, Rh28 cells expressing H-RAS mutants were cultured in differentiation media and stained for the myogenic marker sarcomere myosin using the anti-sarcomere-myosin hybridoma MF20. Representative images are shown (magnification ×100). Cells expressing oncogenic H-RAS show increased staining. However, when compared to the positive control HSMMs, cells expressing oncogenic RAS did not display the same degree of elongation and myotube formation. HSMM, human skeletal muscle myoblasts.

Having observed that oncogenic RAS was detrimental to Rh28 cell growth, we continued to investigate the etiology of the growth inhibition. We first explored the possibility of increased myogenic differentiation. Under light microscopy of standard growth culturing conditions, Rh28 cells expressing vector, wild type RAS, or dominant negative RAS all displayed normal morphology of small round cells, whereas cells expressing oncogenic RAS displayed an elongated morphology (Fig. 3A). To assess for myogenic differentiation, we cultured the cells in myogenic differentiation-permissive conditions for five days and stained them for the myogenic marker MF20. Compared to the other groups, Rh28 cells expressing oncogenic RAS displayed a decreased cell density and an increase in overall staining for MF20, consistent with myogenic differentiation (Fig. 3B). However, when compared to the positive control HSMMs, cells expressing oncogenic RAS did not display the same degree of elongation and myotube formation, suggesting that while oncogenic RAS caused an increase in differentiation markers, it was not the sole mechanism of growth inhibition.

### Expression of oncogenic HRAS induces senescence in Rh28 aRMS cells

**Figure 4:**
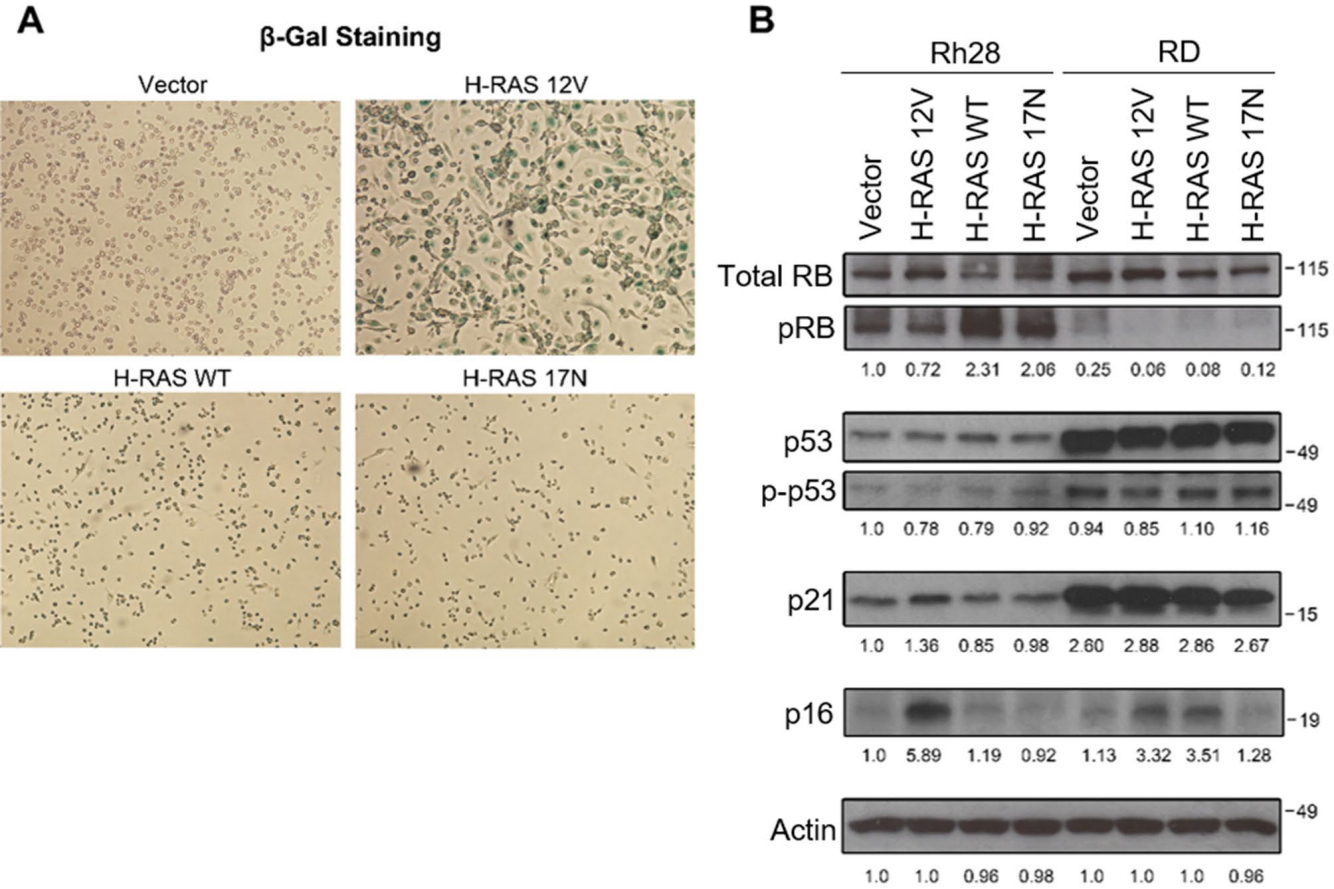
Expression of oncogenic H-RAS induces senescence in Rh28 aRMS cells. (**A**) To assess senescence, Rh28 cells expressing H-RAS mutants were stained for β-gal. Representative images are shown (magnification ×100). Expression of oncogenic H-RAS increased β-gal staining whereas the other H-RAS mutants did not. (**B**) Expression of RB and p53 pathway members was examined by immunoblot in both Rh28 and RD cells expressing H-RAS mutants. Representative blots are shown. While the expression of oncogenic H-RAS led to inconsistent changes in the levels of total RB, total p53, and phospho-p53 over multiple replicates, p16 and p21 were consistently elevated and pRB levels were decreased with the expression of oncogenic H-RAS in aRMS Rh28 cells; this effect was not seen to the same degree in R-eRMS RD cells. Actin used as a loading control. Densitometry quantitation shown below blots and is normalized to Rh28 Vector control.

Based on the literature surrounding RAS and OIS, we turned our attention to the possibility of increased senescence as the source of growth arrest. In addition to elongating, Rh28 cell populations expressing oncogenic RAS also underwent other morphologic changes including enlargement and flattening, morphologic hallmarks of senescence. Therefore we stained the cells for β-galactosidase, a biochemical indicator of senescence. We found that while the vector, wild type RAS, and dominant negative RAS cells remained β-gal negative, the oncogenic RAS cells stained positive as indicated by their blue color (Fig. 4A). Since both the p16INK4a-RB pathway and ARF-p53 pathway have been implicated as crucial mediators of senescence^44–46^, we examined the expression of RB and p53 pathway members via immunoblot in Rh28 and RD cells stably expressing RAS mutants. While the expression of oncogenic RAS in Rh28 cells led to moderate and inconsistent changes in the levels of total RB, total p53, and phospho-p53 over multiple replicates, the levels of p16 and p21 were consistently elevated, and phospho (inactive) RB levels consistently decreased with oncogenic RAS expression (Fig. 4B, Supplementary Fig. S5). This effect was not seen to the same degree in RD cells, although there was a modest increase in p16 in RD cells expressing H-RAS 12V or H-RAS WT, so that despite the lack of change in the growth curve, at least some of the RD cell population was responding at a signaling level to increased RAS activity. In sum, these data suggest that oncogenic RAS expression induces senescence in Rh28 aRMS cells and is associated with p16 and p21 upregulation and RB tumor suppressor activation.

### Expression of constitutively active AKT or ERK also impairs Rh28 aRMS cell growth

**Figure 5:**
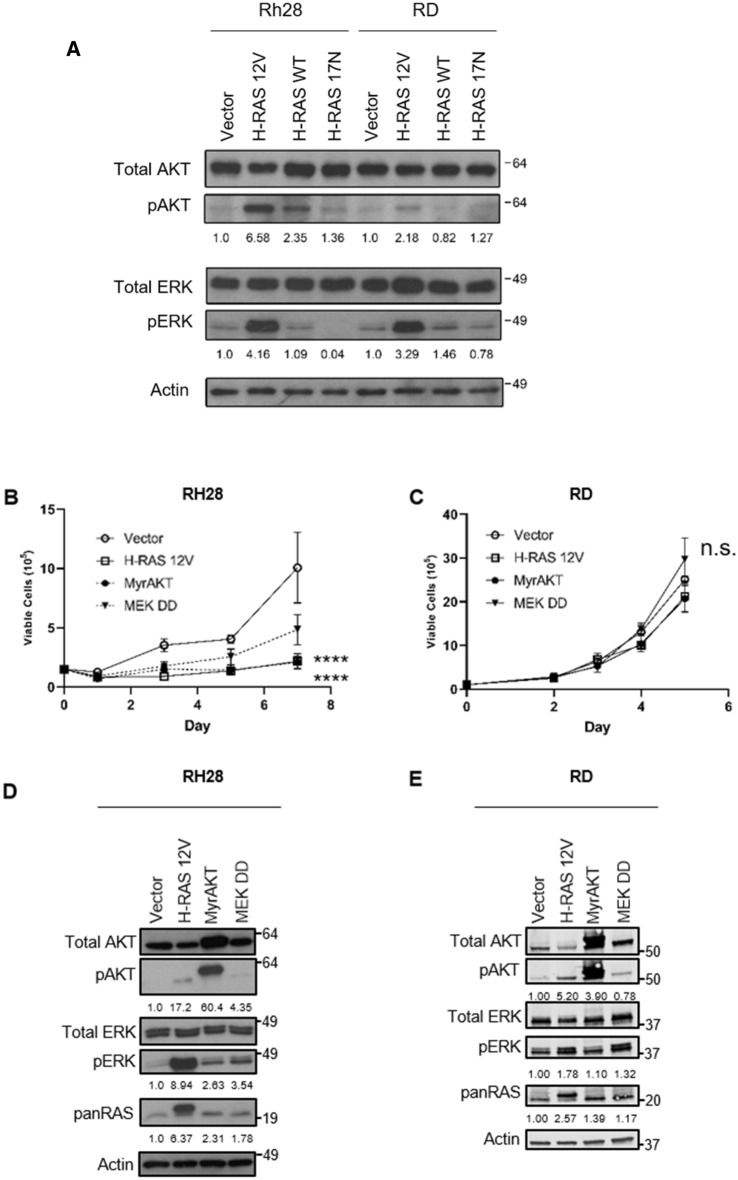
The growth inhibitory effect of oncogenic H-RAS in Rh28 aRMS cells is mediated by the AKT and ERK pathways. (**A**) Expression of RAS effector pathway members (AKT and ERK) was examined by immunoblot in Rh28 and RD cells ectopically expressing RAS mutants. The levels of pAKT increased with the expression of oncogenic RAS in Rh28 but not in RD, whereas the levels of pERK increased with oncogenic RAS in both Rh28 and RD. (**B**) Expression of activated AKT in Rh28 cells caused growth inhibition comparable to oncogenic RAS, whereas activated MEK led to cell growth inhibition, but not as much as activated AKT or oncogenic RAS. (**C**) RD cell population growth was unaffected by expression of the activating mutants. (**D**) Validation of expression of RAS effector activating mutants (MyrAKT, MEK DD) in Rh28 cells by immunoblot. (**E**) Validation of expression of RAS effector activating mutants (MyrAKT, MEK DD) in RD cells by immunoblot. Actin used as a loading control. For (**A**), (**D**) and (**E**), densitometry quantitation is shown below blots and is normalized to Vector control. (*p < 0.05; **p < 0.01; ****p < 0.0001).

Next, we moved downstream to examine the RAS effector pathways involved in OIS in Rh28 aRMS cells. We first analyzed the levels of AKT and ERK activity in Rh28 and RD cells stably expressing RAS mutants by immunoblotting for total and phospho (active) AKT and ERK. Interestingly, the levels of pAKT markedly increased with the expression of oncogenic RAS in Rh28 but less in RD, whereas the levels of pERK increased with oncogenic RAS in both Rh28 and RD (Fig. 5A, Supplementary Fig. S6). To functionally assess these RAS effector pathways, we expressed activating mutants of single RAS effector pathways in Rh28 cells and measured cell growth over seven days. Expression of myristoylated (activated) AKT led to growth arrest that was comparable to oncogenic RAS. Expression of activated MEK also led to growth inhibition, though not to the same degree as oncogenic RAS and MyrAKT (Fig. 5B). Similar to that seen with the RAS mutant studies, RD cells were agnostic in their population growth to RAS effector pathway mutants (Fig. 5C). These activating mutants (MyrAKT, MEK1DD) have been well documented to increase phosphorylation of their target pathways in the case of AKT and MEK^47–49^, which we confirmed using immunoblot (Fig. 5D–E, Supplementary Figs. S7, S8). As assessed by densitometry, in this experiment the H-RAS V12 mutant was also effective in activating all of the downstream RAS effector pathways, as assessed by increased phospho-AKT and phospho-ERK (Fig. 5D). Interestingly, pan-RAS protein levels increased slightly in the cells expressing the activating mutants even though the pathways activated were downstream of RAS, suggesting a feedback loop. In sum, these data suggest that the AKT and ERK RAS effector pathways impair Rh28 cell growth when ectopically overexpressed and could be the trigger for OIS.

### The RAS wild-type eRMS cell line RMS-YM is also growth-inhibited in response to oncogenic HRAS expression

**Figure 6:**
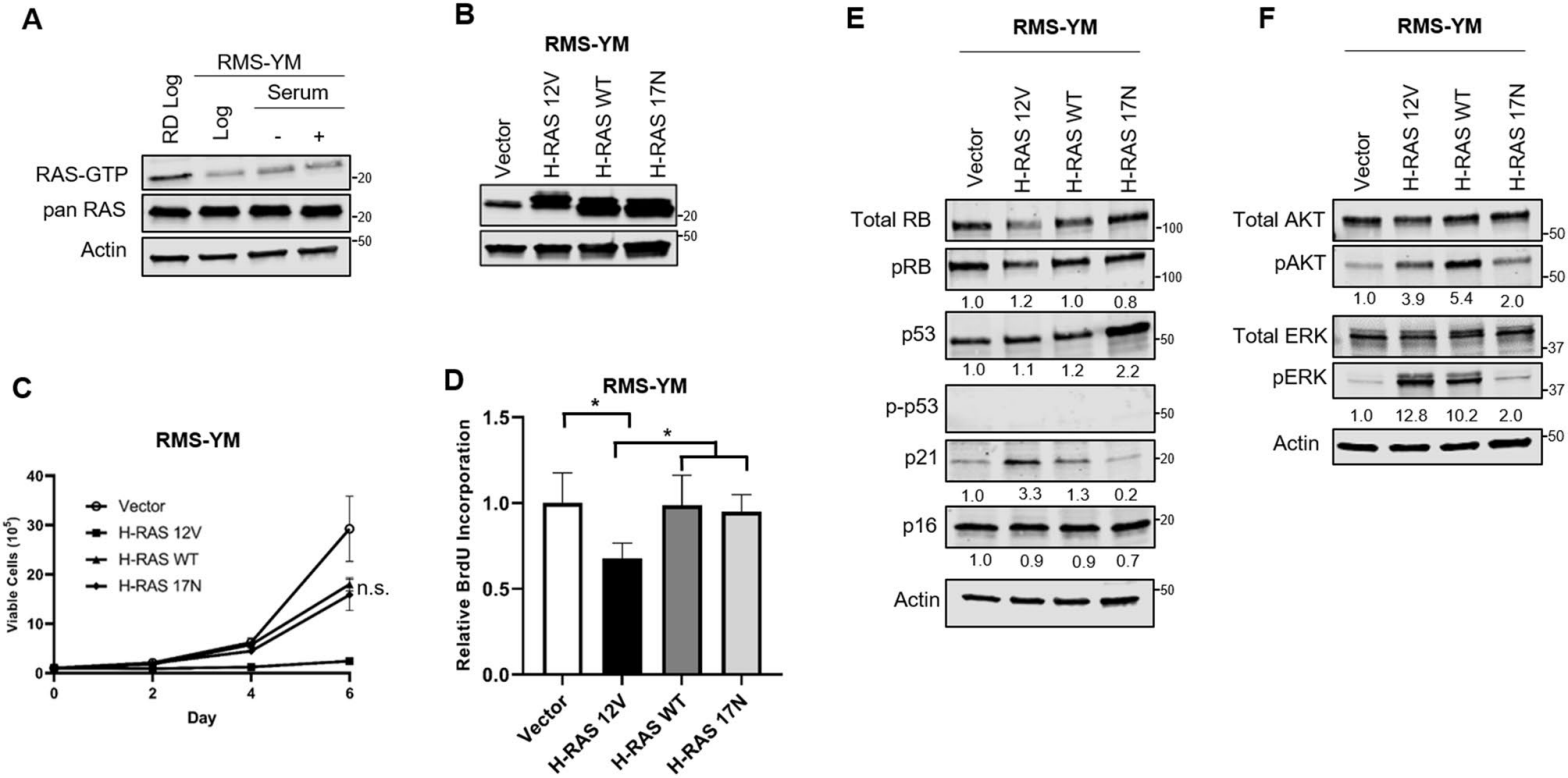
Stable expression of oncogenic H-RAS 12V inhibits growth and decreases proliferation in RAS wild type eRMS cells. (**A**) Under log phase growth conditions, the RAS WT eRMS cell line, RMS-YM, displayed lower levels of RAS-GTP than the *N-RAS* mutant RD cell line. Under serum stimulation conditions, RAS-GTP levels were still lower in RMS-YM cells than RD cells. Cells were collected either in log phase, after 48 h of serum starvation, or 48 h of serum starvation followed by 1 h of growth in serum media. RD cells collected in log phase were included as a positive control. (**B**) Immunoblot confirmation of ectopic expression of H-RAS 12V, H-RAS WT, or H-RAS 17N in RMS-YM cells. (**C**) Expression of oncogenic H-RAS caused growth suppression in RMS-YM cells. Wild type and dominant negative H-RAS also impaired growth in RMS-YM cells, though not to the same degree as oncogenic H-RAS, and did not meet statistical significance. (**D**) RMS-YM cell proliferation decreased in response to expression of oncogenic H-RAS, as measured by BrdU assay. (**E**) Whereas H-RAS 12V and H-RAS WT drove upregulation of p16 in Rh28 and RD cells with modest increase in p21 in Rh28, RMS-YM cells showed upregulation of p21 with no change to p16. Expression of dominant negative H-RAS led to a two-fold increase of p53. (**F**) Expression of H-RAS 12V and H-RAS WT constructs increased AKT and ERK activation as assessed by levels of pAKT and pERK. Actin used as a loading control. (*p < 0.05; **p < 0.01; ****p < 0.0001).

To further explore our original hypothesis that eRMS cell lines tolerate oncogenic RAS while aRMS cell lines do not, we examined the impact of oncogenic HRAS and activating RAS effector mutants in RAS wild type RMS cells, with the prediction that as an eRMS cell type, it would tolerate oncogenic RAS expression. We first examined RAS levels in RMS-YM cells compared with the R-eRMS RD cell line (Fig. 6A, Supplementary Fig. S9). While total RAS expression was similar between the two cell lines, RAS-GTP levels were lower in log phase RMS-YM cells, and similar to Rh28 cells, could not be significantly stimulated after 48 h serum starvation. We then, as before, ectopically overexpressed oncogenic HRAS (H-RAS 12V), wild type HRAS (H-RAS WT), or dominant negative HRAS (H-RAS 17N) in RMS-YM cells, and expression was again confirmed via immunoblot using a pan-RAS antibody (Fig. 6B, Supplementary Fig. S10). Unexpectedly, both wild type and dominant negative HRAS moderately impaired the growth of RMS-YM cells (although not statistically significant), while expression of oncogenic HRAS caused growth arrest (Fig. 6C) and inhibition of proliferation as assessed by BrdU incorporation (Fig. 6D). To mirror the prior studies in the Rh28 and RD cells, we examined the impact of the RAS mutants in the RMS-YM cells on the p53, RB, AKT and ERK pathways. H-RAS 12V and H-RAS WT effectively activated p21, while dominant negative H-RAS 17N led to increased p53 expression (Fig. 6E, Supplementary Fig. S11). H-RAS 12V and H-RAS WT both activated the AKT and ERK pathways (Fig. 6F, Supplementary Fig. S12). Overall, RMS-YM cells appear to have low expression of p53, especially compared to RD cells that harbor *TP53* mutations^32^ (Supplementary Fig. S13), with Ser15 p-p53 below the limit of detection on our blots. This is likely due to the described *MDM2* amplification in RMS-YM cells^50^.

**Figure 7:**
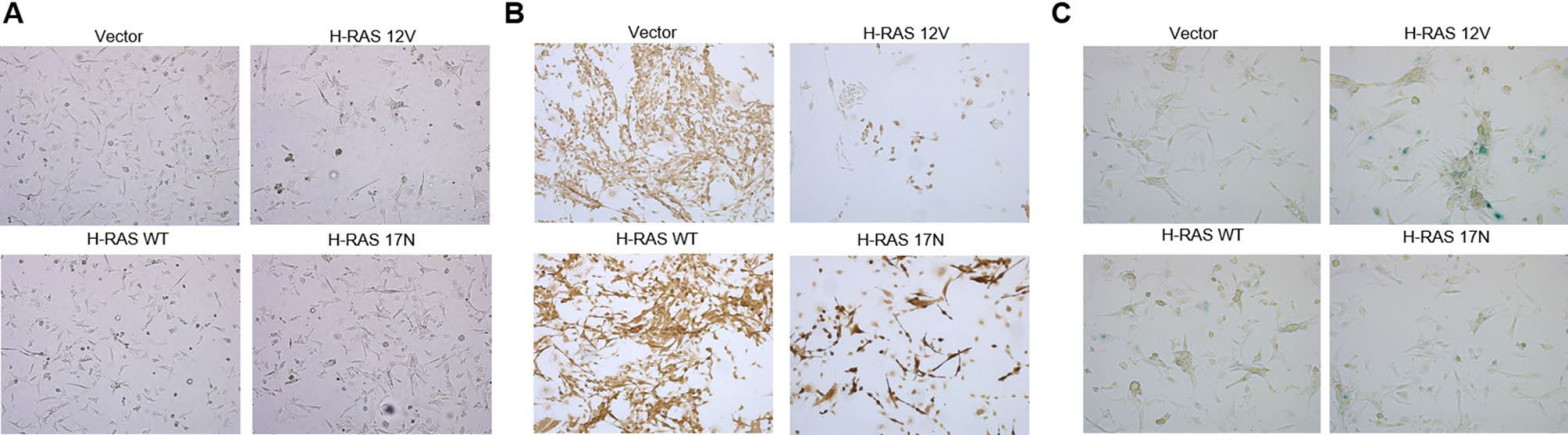
Analysis of morphologic and phenotypic changes of RMS-YM cells in response to ectopic RAS expression. (**A**) Expression of H-RAS constructs did not induce significant morphology changes as observed under light microscopy, despite slow growth kinetics. (**B**) To assess for markers of differentiation, RMS-YM cells expressing H-RAS mutants were cultured in differentiation media and stained for the myogenic marker sarcomere myosin using the anti-sarcomere-myosin hybridoma MF20. Representative images are shown. Cells expressing oncogenic H-RAS are not viable after multiple days in low serum conditions whereas cells expressing dominant negative H-RAS have become elongated and show increased staining. (**C**) To assess senescence, RMS-YM cells expressing H-RAS mutants were stained for β-gal. Representative images are shown. Expression of oncogenic H-RAS increased β-gal staining (blue color) whereas the other H-RAS mutants did not.

**Figure 8:**
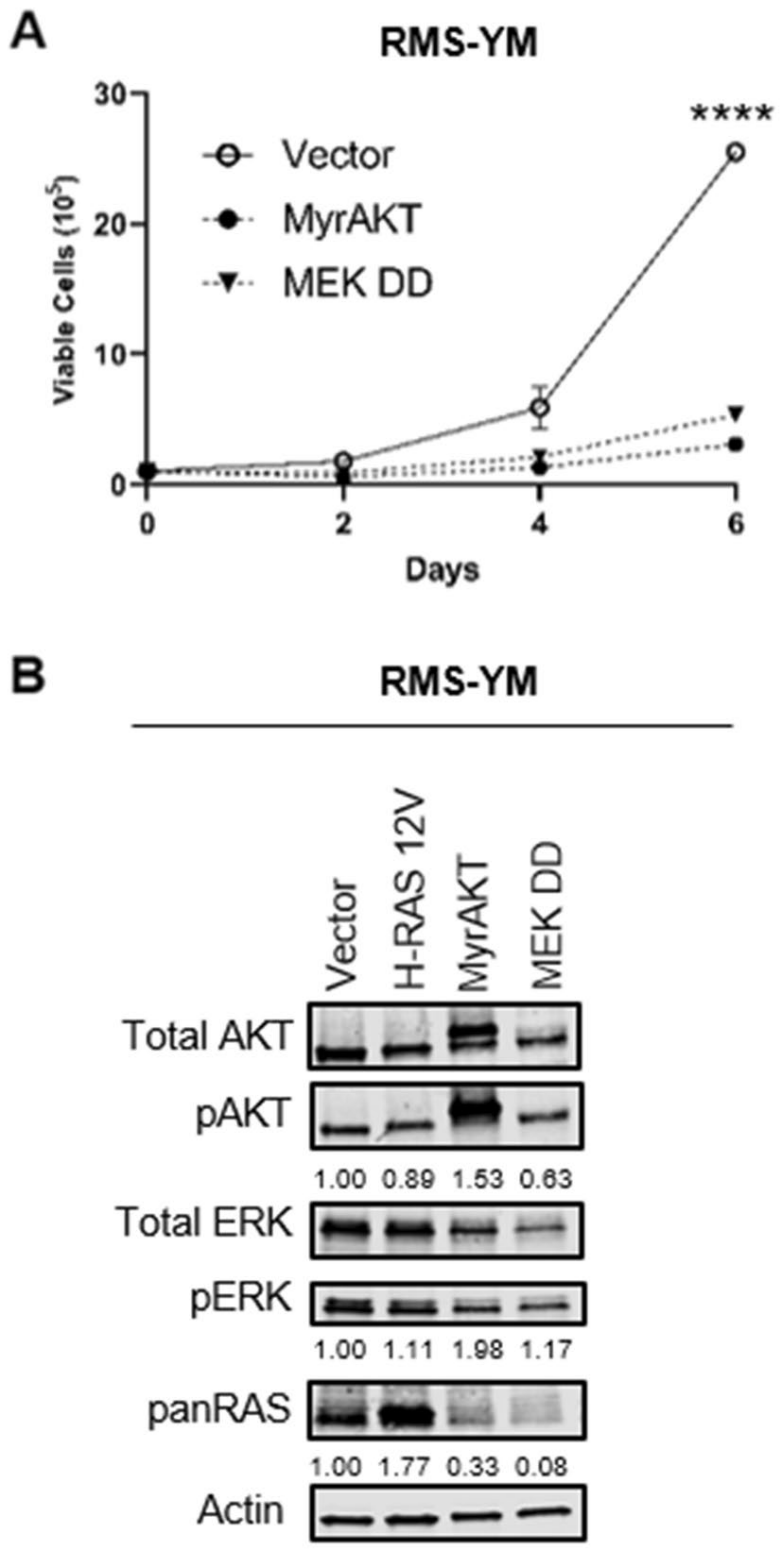
The growth inhibitory effect of oncogenic H-RAS in the RAS wild type eRMS cell line RMS-YM is mediated in part by the AKT and ERK pathways. (**A**) Stable expression of activated RAS effector mutants (activated MyrAKT or activated MEK DD) is deleterious to RMS-YM cell growth compared to vector control, as assessed by manual cell counting over time. (**B**) Immunoblot to assess expression of RAS effector mutants in RMS-YM cells shows a robust increase in AKT activation in MyrAKT-expressing cells and a modest increase in ERK activation in MEK DD-expressing cells. Actin used as loading control. (*p < 0.05; **p < 0.01; ****p < 0.0001).

The growth arrest caused by oncogenic H-RAS 12V did not lead to as stark a morphology alteration in RMS-YM cells (Fig. 7A) when compared to the Rh28 cells. In myogenic assays, they were largely non-viable after multiple days in differentiation conditions (Fig. 7B). But similar to that seen in Rh28 cells, the RMS-YM cells expressing oncogenic H-RAS did exhibit increased senescence as assessed by β-galactosidase staining (Fig. 7C). Interestingly, RMS-YM H-RAS 17N cells, which grew comparably to RMS-YM cells ectopically expressing H-RAS WT under normal growth conditions, seemed sparser after the five-day differentiation period and showed increased myogenic differentiation as assessed by MF20 staining (Fig. 7B). Finally, similar to that seen in the Rh28 cells, ectopic overexpression of constitutively active AKT or MEK constructs impaired RMS-YM cell growth (Fig. 8A). A noticeable difference was that forced expression of these RAS effector mutants was associated with a decrease in overall RAS protein levels (Fig. 8B, Supplementary Fig. S14), perhaps suggesting that the RMS-YM cells are trying to restore equilibrium in RAS signaling. All together, these data indicate that RMS-YM RAS wild-type eRMS cells respond to changes in oncogenic HRAS expression in a manner more similar to aRMS than R-eRMS and predict that eRMS tumors can be further sub-classified by mutational profile.

## Discussion

Although *RAS* mutations are common in eRMS, it has been unclear why aRMS cells bearing PAX3-FOXO1 fusions rarely harbor *RAS* mutations. We reasoned that either oncogenic RAS is not required for tumorigenesis of aRMS or is not tolerated in aRMS. In this study, we show that forced expression of HRAS triggers OIS in PAX3-FOXO1-positive Rh28 aRMS cells, and this senescence is associated with increased p16, p21, and RB pathway activity. It is important to note that we only studied the effect of HRAS. NRAS and KRAS will need to be examined independently, and this will be supported by RAS isoform-specific antibodies^51^. Given the observations that *H*, *N*, and *KRAS*-mutant eRMS are associated with specific age ranges and anatomic locations, this will contribute to the understanding of the role of dysregulated development in R-eRMS^52^.

Though OIS has been observed in pre-malignant cells from a variety of tissues^13–18^, there are relatively few examples of activation of OIS in cancer cells. In non-small cell lung cancer cell lines harboring *KRAS* mutations, latent OIS can be reactivated when another oncogene, *TWIST1*, is silenced^53^. In a mouse model of lung cancer, OIS occurred when oncogenic *KRAS* and *BRAF* were expressed concurrently^54^. These examples reveal the mutually exclusive nature of some oncogene combinations, such as *KRAS* and *BRAF,* in human tumors, and we posit that this mutually exclusive relationship may exist between *HRAS* and *PAX3-FOXO1*. Interestingly, when oncogenic *RAS* has been expressed ectopically in cell lines concurrently with *PAX3-FOXO1*, it has been in the setting of deficient p53 activity, resulting from prior *TP53* mutations such as in Rh30 cells or acquired through expression of SV40 T antigen or dominant negative p53 constructs^55–57^. When oncogenic *NRAS* was detected in PAX3-FOXO1-positive aRMS tumor tissue, it was accompanied by loss of heterozygosity of chromosome 11p^58^. It remains to be seen whether other genetic changes influence the concurrence of *RAS* and *PAX3-FOXO1* expression in aRMS. It is important to note that while an excess of RAS signaling was not tolerated in Rh28 aRMS cells, deficient RAS signaling was also detrimental. This is shown by the effect of dominant negative HRAS in the Rh28 cells, and others have also recently found that impaired RAS signaling suppresses aRMS cell and xenograft growth^59,60^. The changes in RAS protein expression we observed in response to ectopic expression of constitutively active RAS effectors in both Rh28 and RMS-YM cells likely reflects the homeostatic feedback loops that occur in RAS signaling^61^.

OIS is a tumor suppressor mechanism that thwarts transformation by activating the p53/RB pathways^12,46,62^. Thus, the very existence of OIS puts selective pressure on tumor cells to acquire mutations in p53/RB pathways to bypass senescence during tumorigenesis. *TP53* mutations can be found in almost every type of cancer, varying in rates from 10% to close to 100%^63^, and *RB* mutations have been found in multiple cancers including retinoblastomas, osteosarcomas, small-cell lung, breast, and cervical carcinomas, and leukemias^64–69^. We show here that OIS in Rh28 cells is associated with increased p16, p21, and RB pathway activation. However, this raises an interesting question—since OIS can be reactivated in an aRMS cell line, does this mean that its RB/p53 pathways are intact? While aRMS tumors have low levels of p53 pathway activity^70^, only a small percentage have mutation or loss of heterozygosity of TP53 compared to eRMS tumors^58^, and some aRMS cell lines with *TP53* mutations are thought to have acquired them after culture in vitro^32^. We speculate that in at least some aRMS cells, p53 and RB pathways are functionally inactivated to permit tumor formation and proliferation, but this functional inactivation is reversible. Our prior data showed that PAX3-FOXO1 cooperates with epigenetic silencing of p16 to bypass senescence^37^. Thus, senescence pathways are turned “off” during tumor formation and growth, but these pathways can be reactivated and turned “on” again by external stressors such as ectopic oncogenic RAS expression. Pro-senescence therapy via p53/RB pathway reactivation is an enticing field of study and may provide new ways to target aRMS tumors^71–74^.

Our study also revealed that in Rh28 cells, hyperactive AKT or ERK signaling inhibits cell growth. While PI3K-AKT signaling is important for cell proliferation and survival in many tumors^75,76^, it is also a known inducer of OIS^21,77,78^. A recent study detected a *PIK3CA* mutation in a PAX3-FOXO1 positive aRMS tumor^79^; however, it is not known whether the mutation was activating or inactivating, making this data difficult to interpret. Also, in our study, the MyrAKT construct we used was not 100% specific for the AKT pathway; though the construct mainly increased pAKT levels, it also increased pERK levels. On the other hand, the MEK1DD construct only increased pERK levels, and it only partially induced growth inhibition. Similar to AKT, hyperactive ERK signaling can also induce OIS^80,81^. It is possible that cooperation between both the AKT and ERK pathways will be needed to fully induce OIS in aRMS, and further studies will be needed to definitively prove the role of these pathways in the senescence phenotype. Future complementary experiments using pharmacologic activators or inhibitors of AKT and MEK/ERK will be useful in this regard.

In summary, we find that oncogenic HRAS expression is detrimental to the growth and leads to OIS in Rh28 aRMS cells. This observation opens the door to future investigation of the mechanisms of OIS in aRMS, including evaluation of other aRMS cell lines, and possibilities for designing aRMS therapies that target reactivation of p53/RB and/or induce senescence. Oncogenic HRAS is similarly detrimental to RMS-YM RAS wild type eRMS cells, distinct from its role as an oncogenic driver in some R-eRMS cells. These data, along with oncogenic RAS isoform studies in RMS^23^, suggest that oncogenic RAS and likely other somatic mutations in eRMS are expressed in a context-dependent manner. The subgroup of eRMS tumors that are RAS pathway wild type or lacking identifiable protein-coding mutations will require further study to understand and therapeutically target their oncogenic drivers.

## Supplementary Information

Supplementary Figures.
