# Supplementary material for: Expression of oncogenic HRAS in human Rh28 and RMS-YM rhabdomyosarcoma cells leads to oncogene-induced senescence
Source: Sci Rep. 2021 Aug 13;11:16505. doi: 10.1038/s41598-021-95355-2 (PMC8363632; doi:10.1038/s41598-021-95355-2)

Expression of oncogenic HRAS in human Rh28 and RMS-YM rhabdomyosarcoma cells leads to oncogene-induced senescence

Supplement

Jenny J. Li[1]*, Alexander R. Kovach[2]*, Margaret DeMonia[2], Katherine K. Slemmons[3], Kristianne M. Oristian[3], Candy Chen[2], Corinne M. Linardic[1,2,3#]

[1]Duke University School of Medicine, Durham, North Carolina, USA
[2]Department of Pediatrics, Duke University School of Medicine, Durham, North Carolina, USA
[3]Department of Pharmacology and Cancer Biology, Duke University School of Medicine, Durham, North Carolina, USA

*These authors contributed equally.

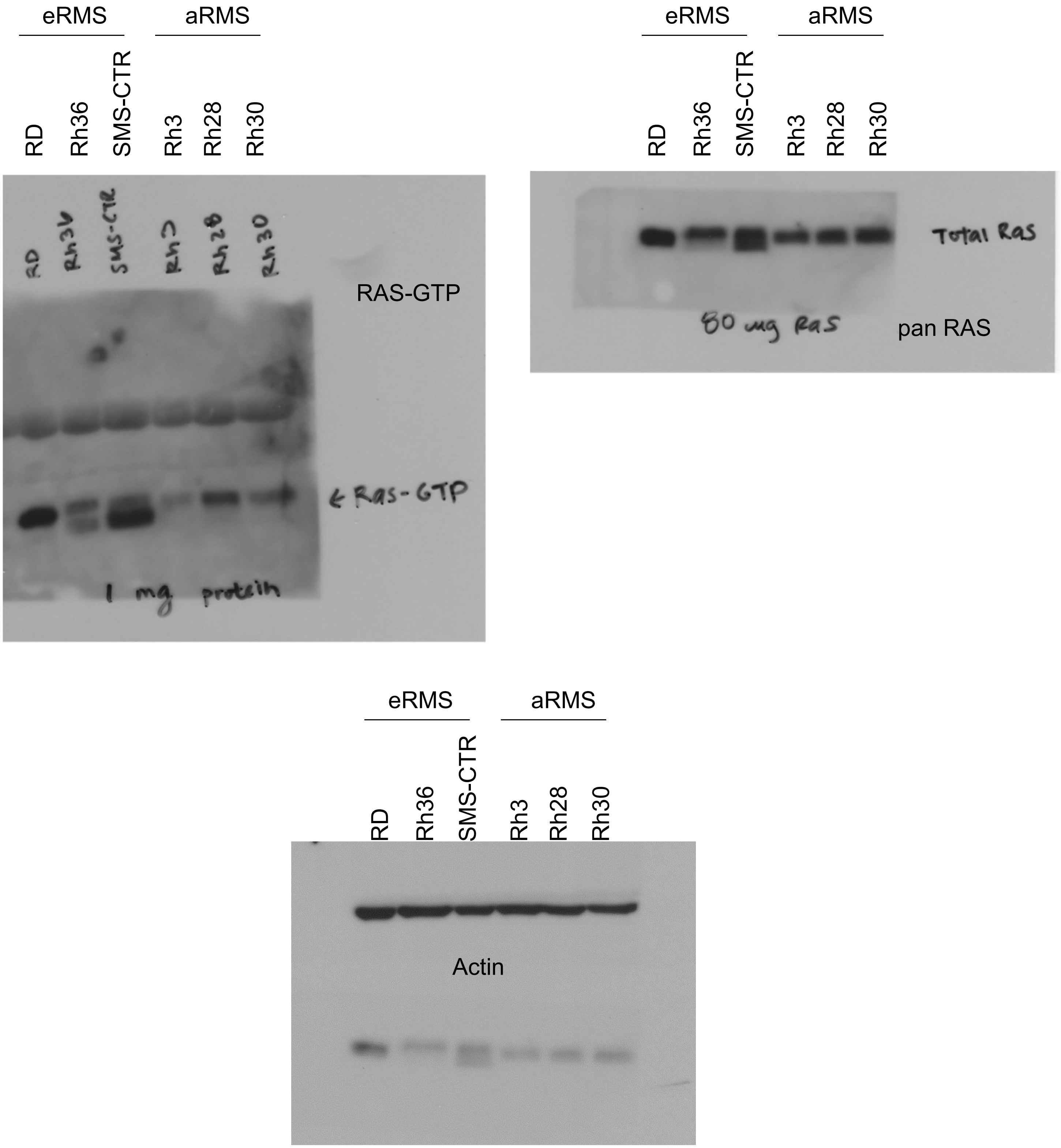

Supplementary Figure 1. Uncropped images of immunoblots corresponding to assembled data presented in Fig.1A. Included here are RAS-GTP, pan RAS, and Actin.

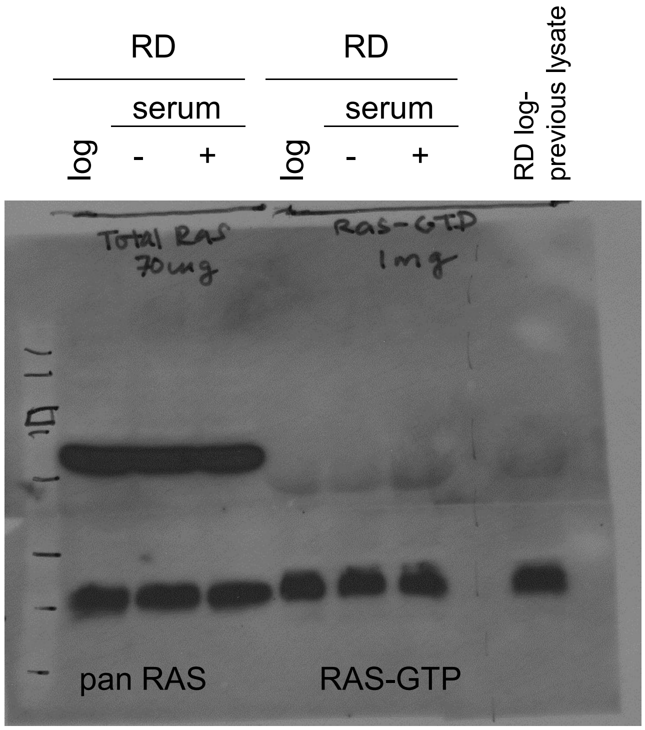

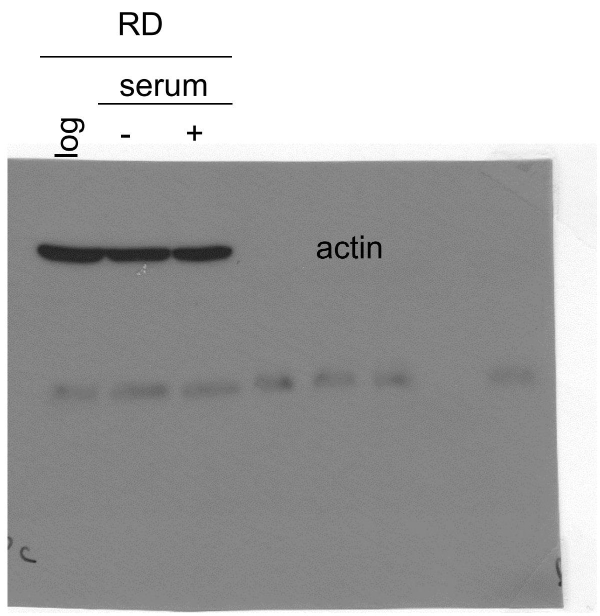

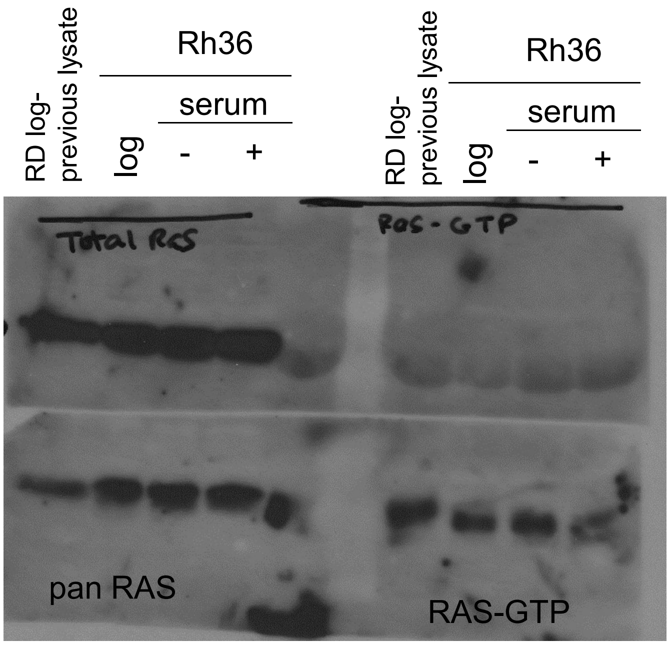

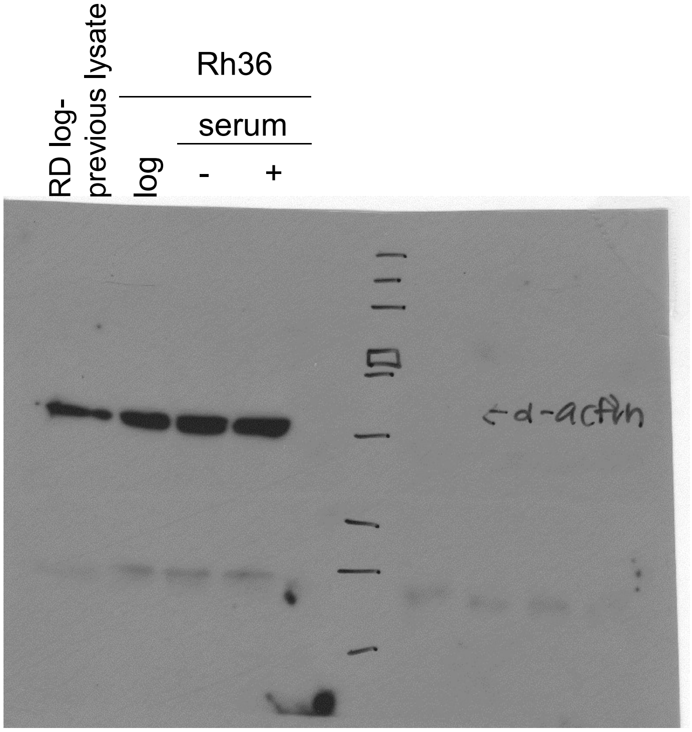

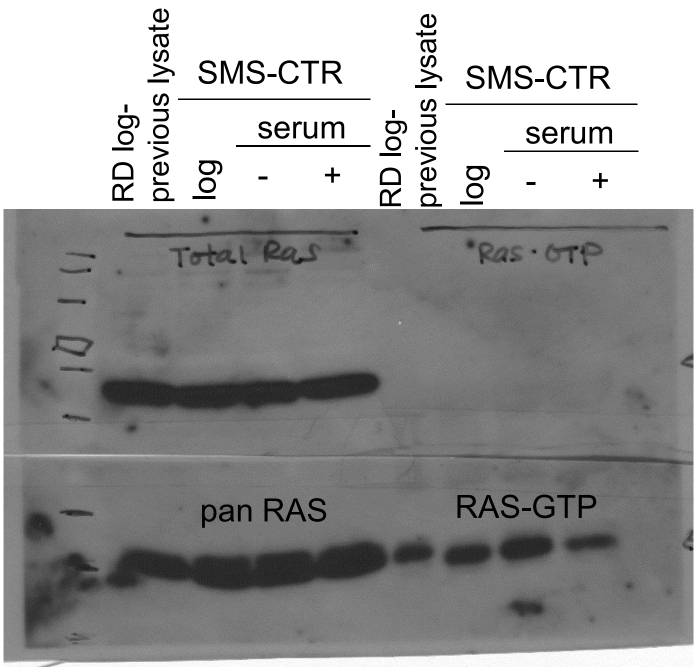

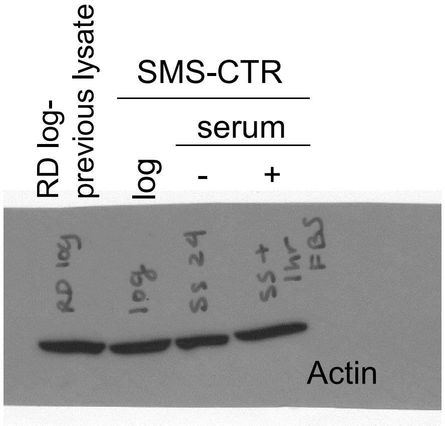

Supplementary Figure 2. Uncropped images of immunoblots corresponding to assembled data presented in Fig.1C. Included here are RAS-GTP, pan RAS, and Actin for eRMS cell lines RD, Rh36 and SMS-CTR.

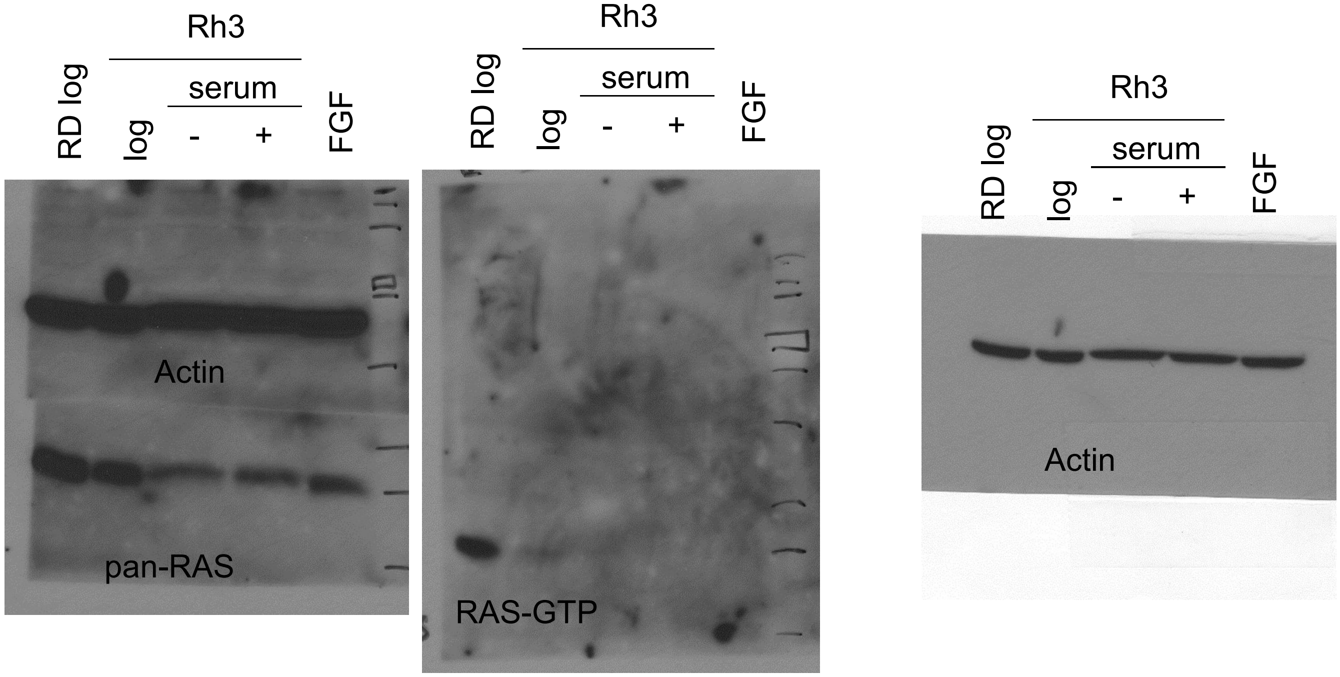

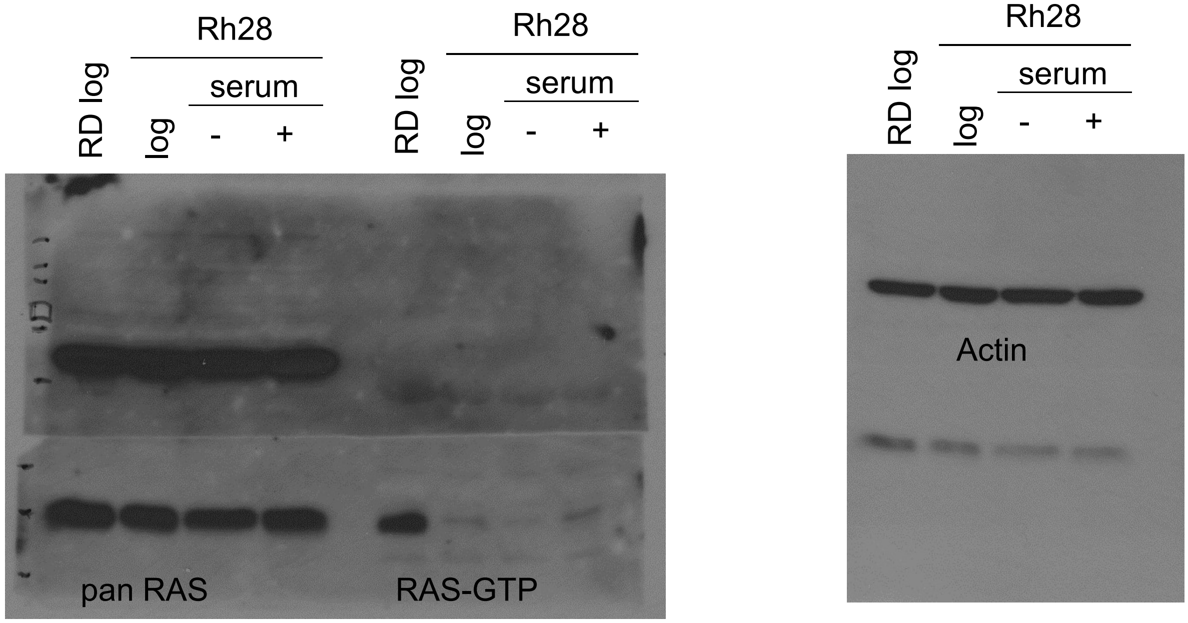

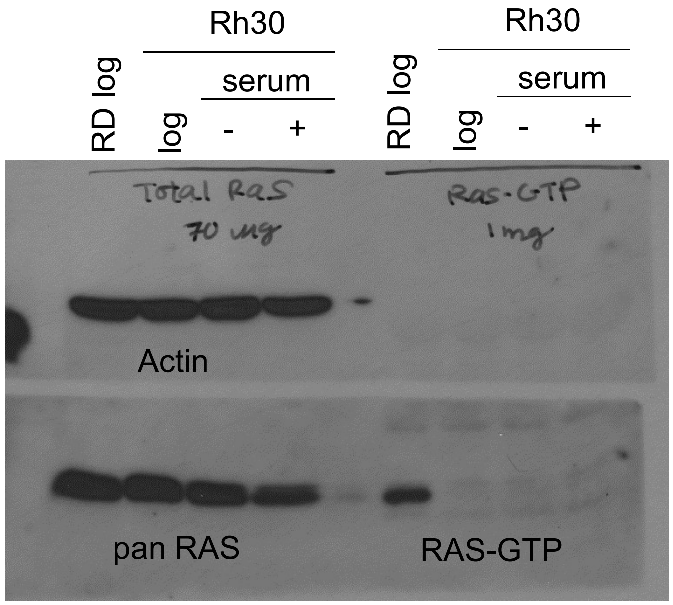

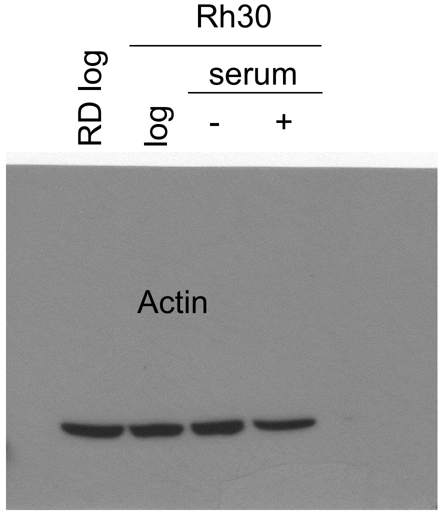

Supplementary Figure 2 (cont'd). Uncropped images of immunoblots corresponding to assembled data presented in Fig.1C. Included here are RAS-GTP, pan RAS, and Actin for aRMS cell lines Rh3, Rh28 and Rh30.

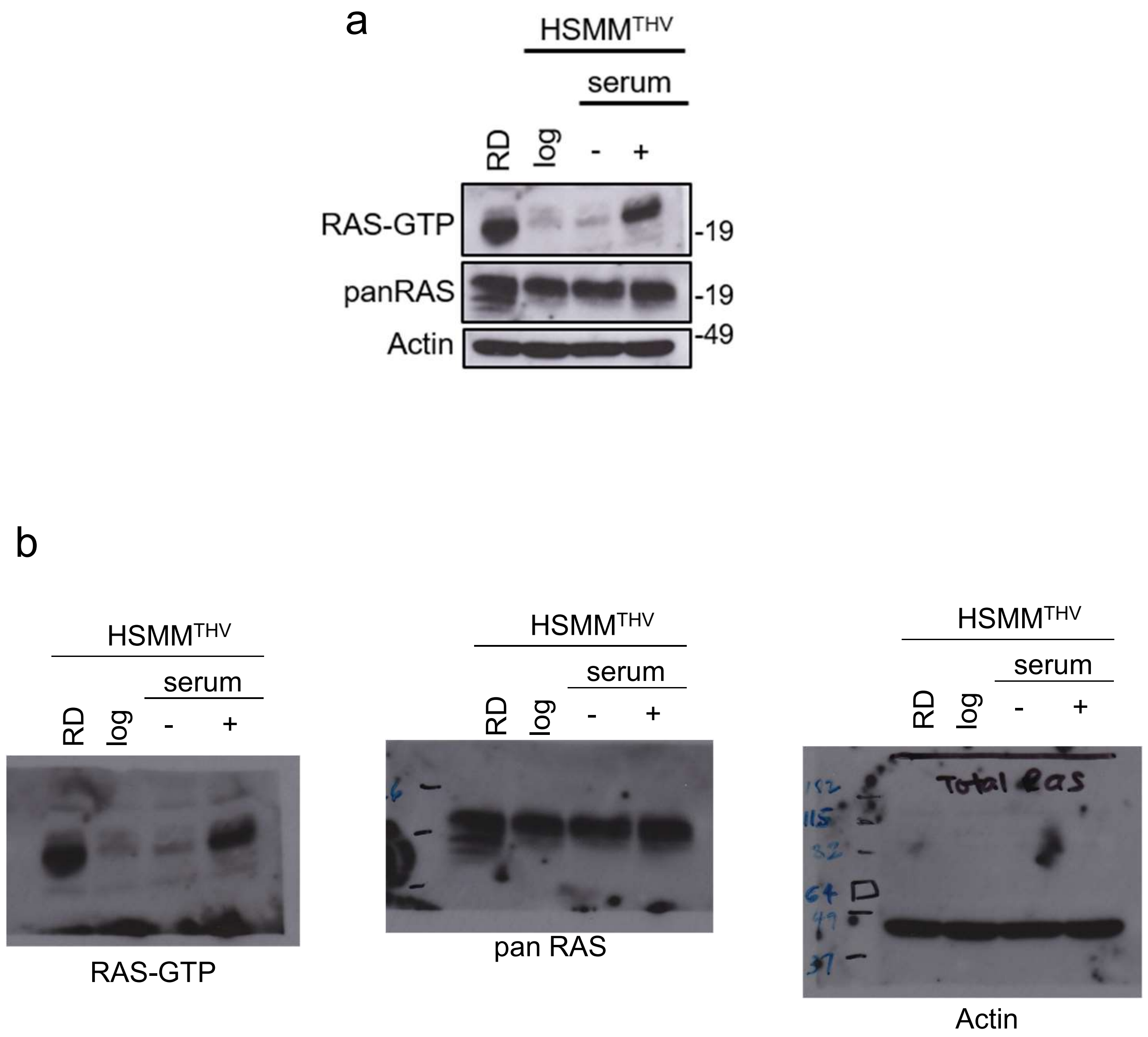

Supplementary Figure 3. Establishing conditions in a control cell line for RAS-GTP pull down with serum starvation. **(a)** RAS-GTP pull down was performed followed by immunoblot with pan RAS antibody. Left to right, RD cells collected from log phase and human skeletal muscle myoblast (HSMM) cells expressing SV40 T/t antigen, hTERT, and empty pBabe vector (THV) collected either in log phase (log), after 48 hours serum starvation (-) or 48 hours serum starvation followed by one hour growth in serum containing media (+). This demonstrates that one hour is sufficient to stimulate robust RAS-GTP expression. **(b)** Uncropped images of immunoblots corresponding to assembled data presented in (a).

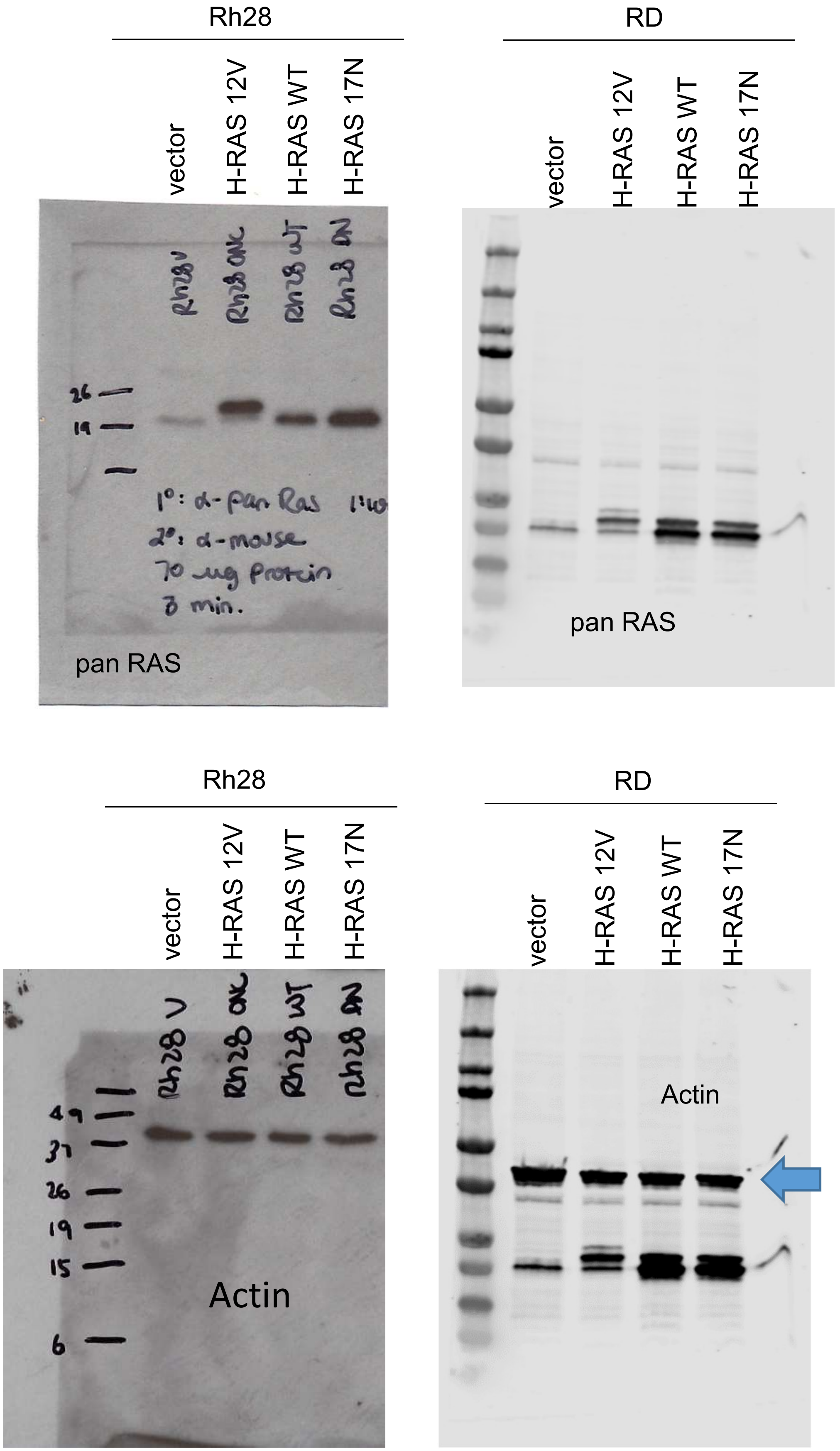

Supplementary Figure 4. Uncropped images of immunoblots corresponding to assembled data presented in Fig.2A. Included here are pan RAS and Actin. Blue arrow indicates relevant Actin band.

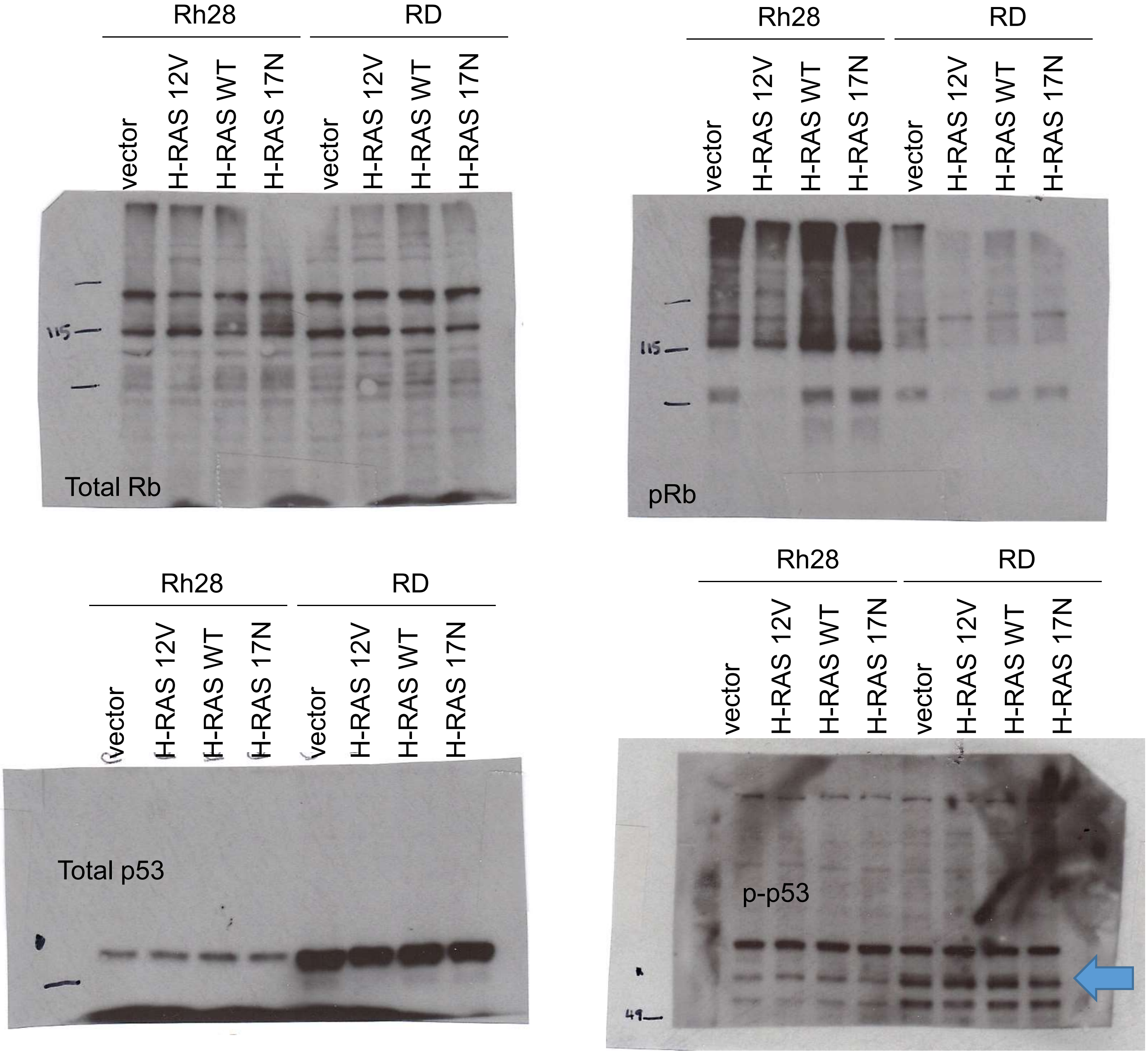

Supplementary Figure 5. Uncropped images of immunoblots corresponding to assembled data presented in Fig.4B. Included here are Total RB, pRB, Total p53, and p-p53. Blue arrow indicates relevant p-p53 band.

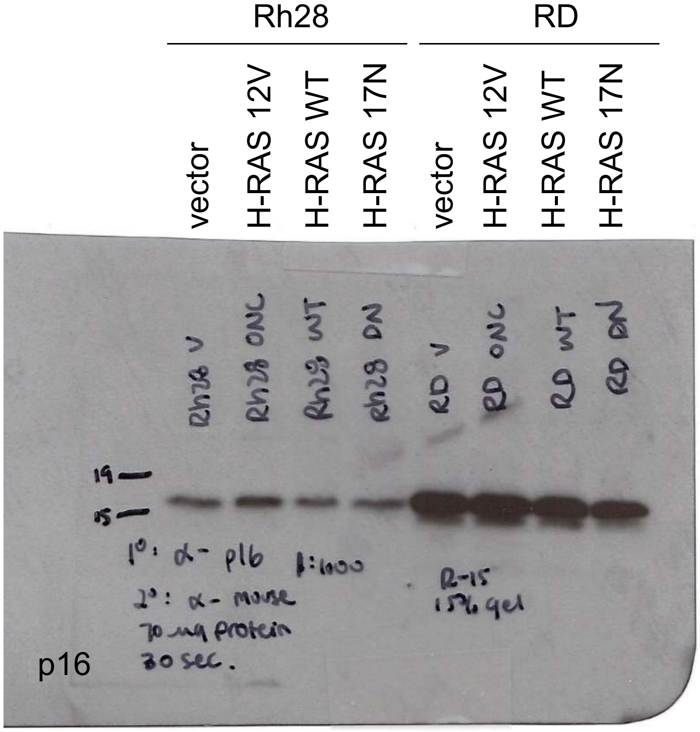

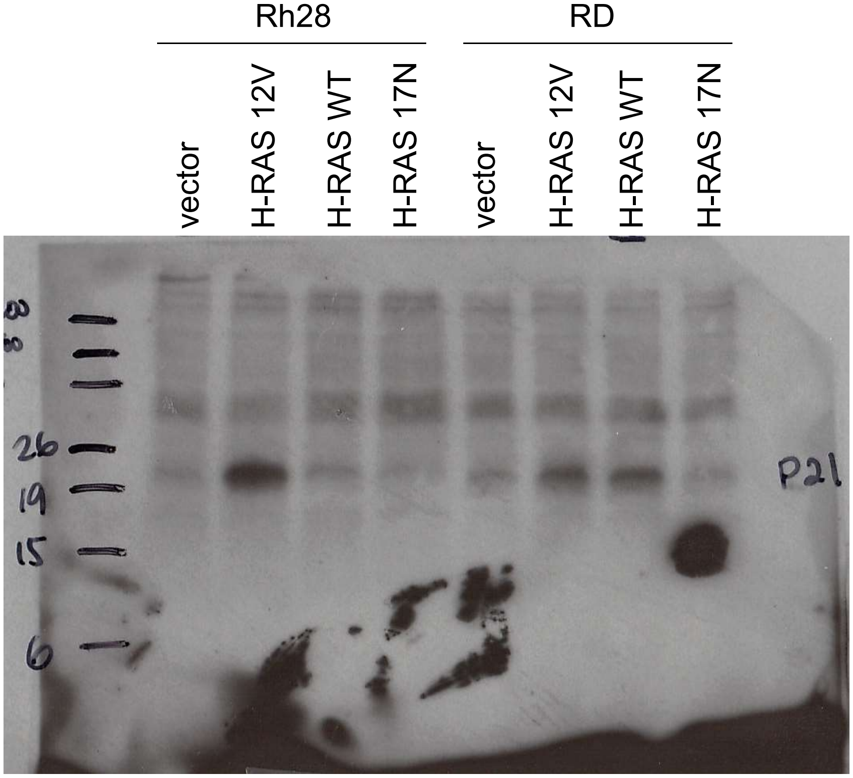

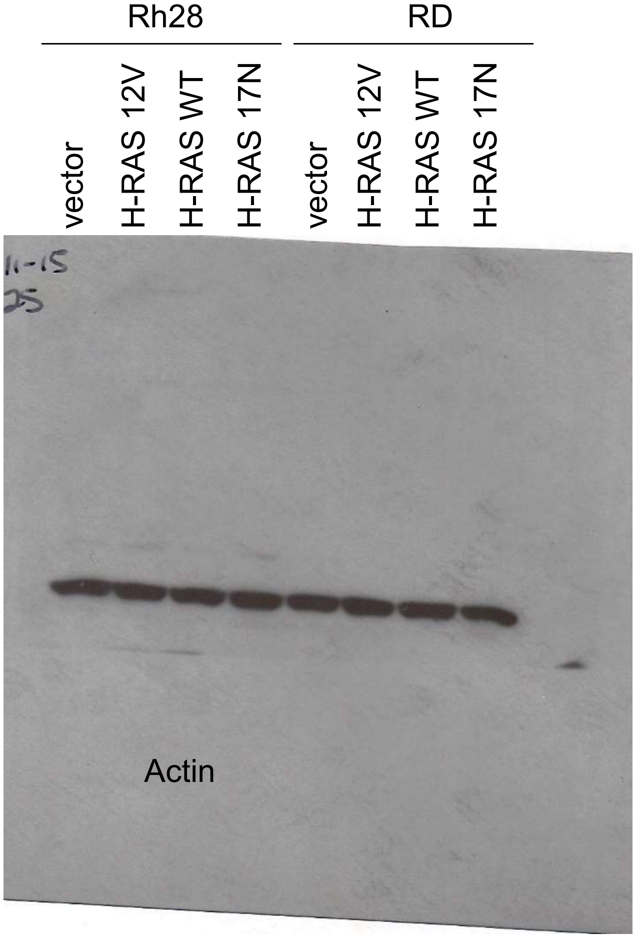

Supplementary Figure 5 (cont'd). Uncropped images of immunoblots corresponding to assembled data presented in Fig.4B. Included here are p16, p21, and Actin.

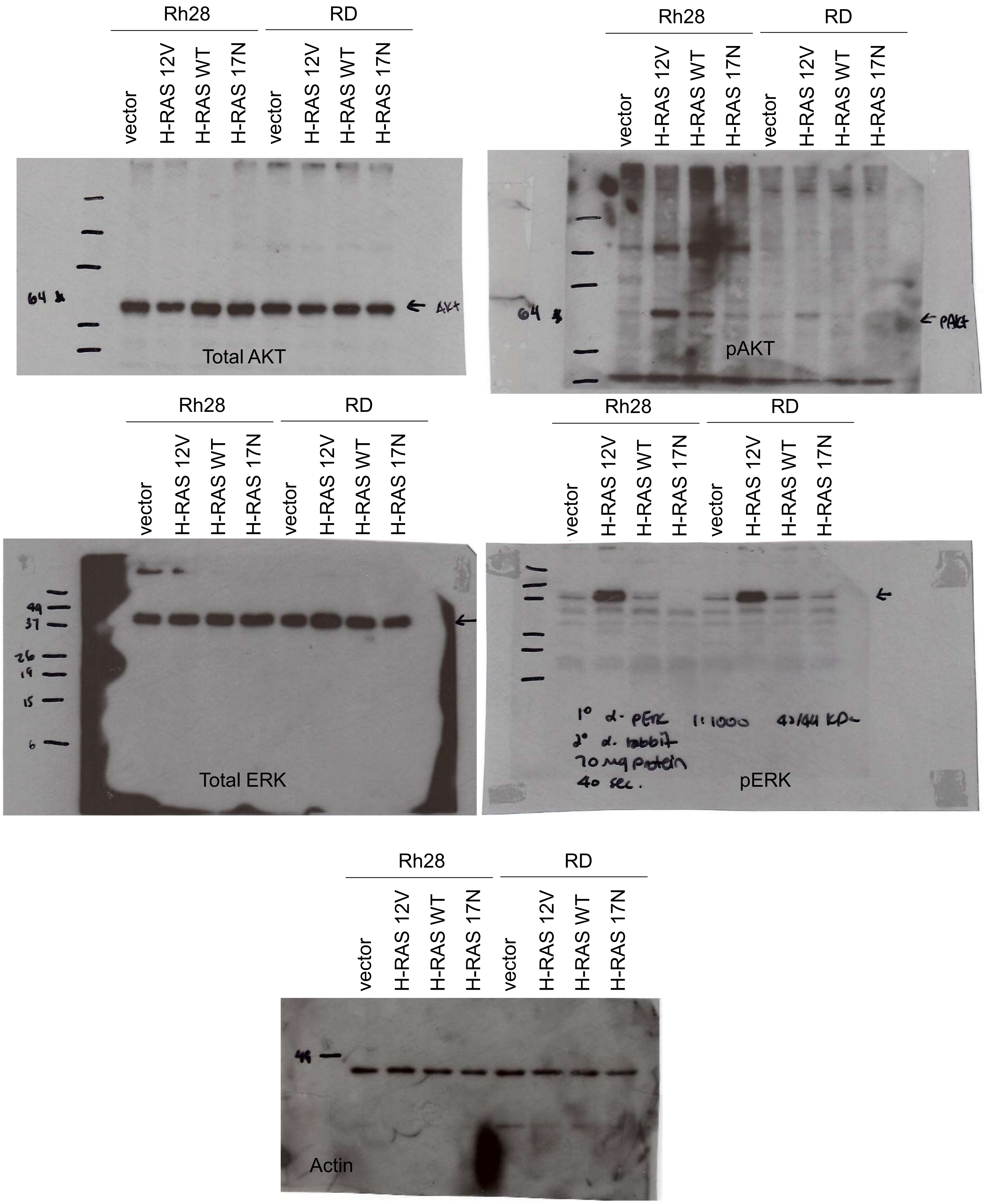

Supplementary Figure 6. Uncropped images of immunoblots corresponding to assembled data presented in Fig.5A. Included here are total AKT, pAKT, ERK, pERK.

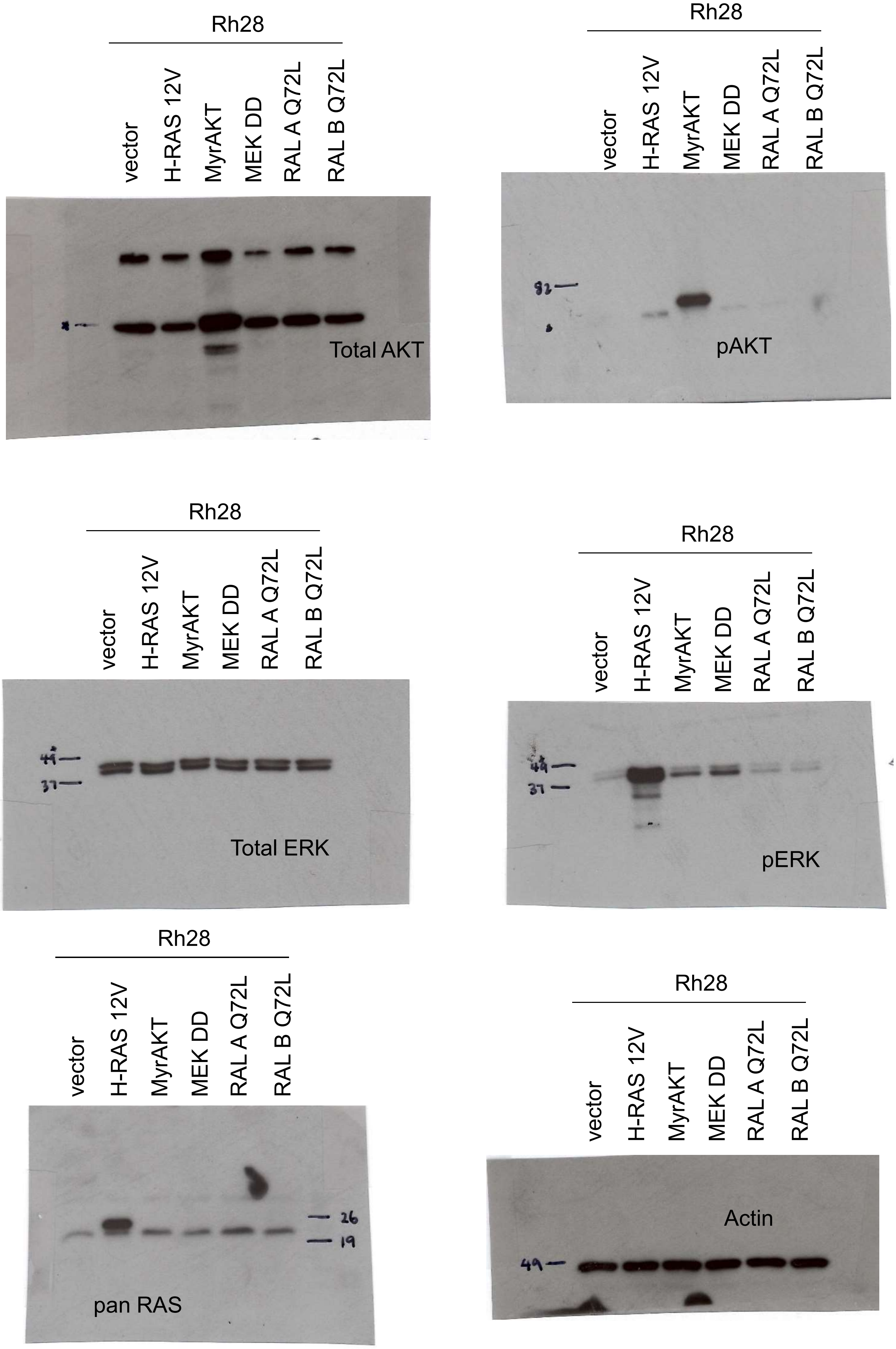

Supplementary Figure 7. Uncropped images of immunoblots corresponding to assembled data presented in Fig.5D. Included here are total AKT, pAKT, ERK, pERK.

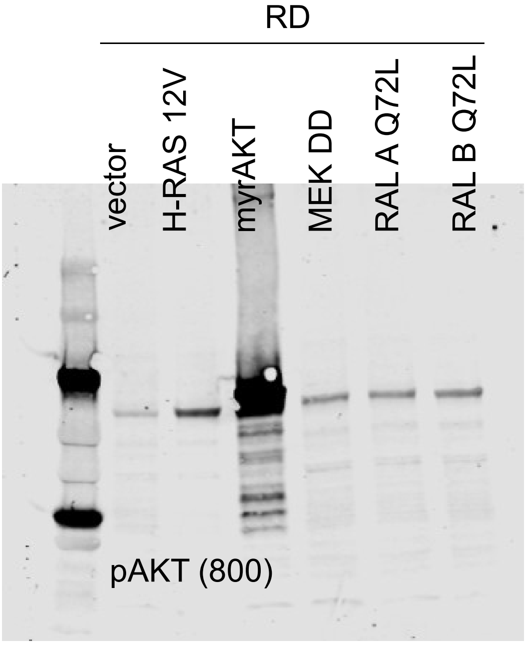

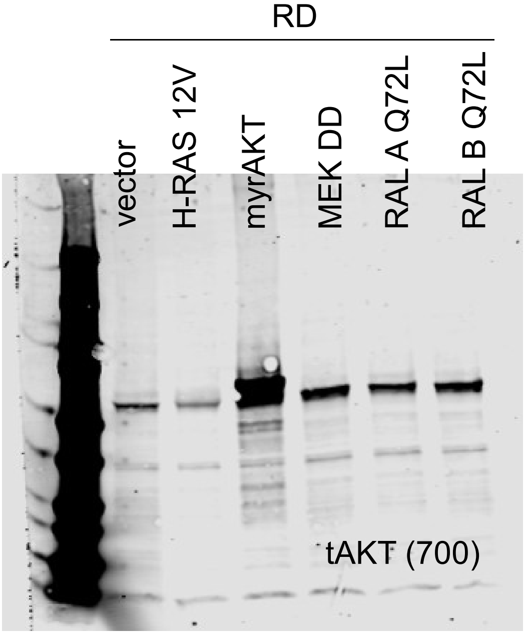

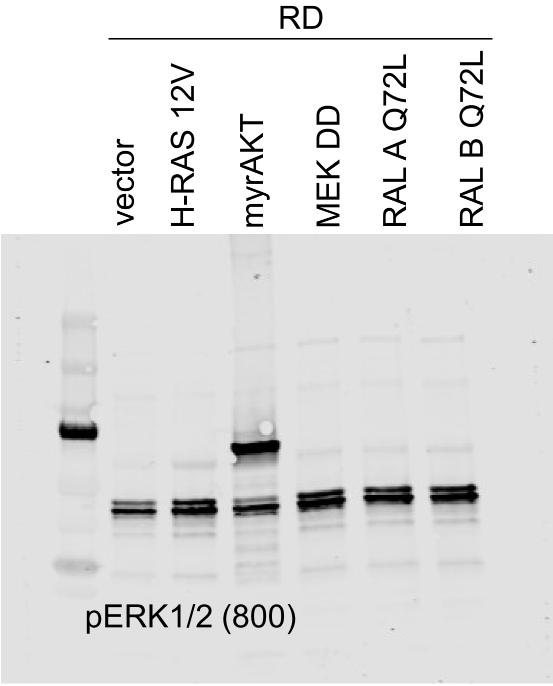

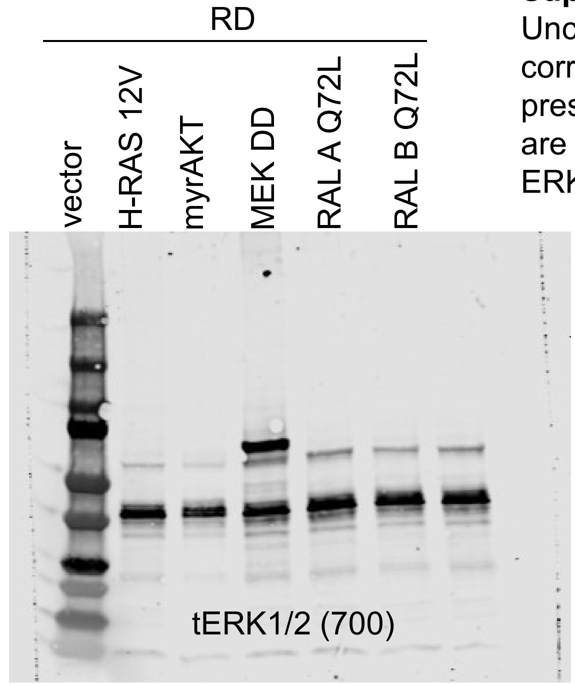

Supplementary Figure 8. Uncropped images of immunoblots corresponding to assembled data presented in Fig.5E. Included here are pAKT, total AKT, pERK1/2, total ERK1/2, pan RAS and Actin.

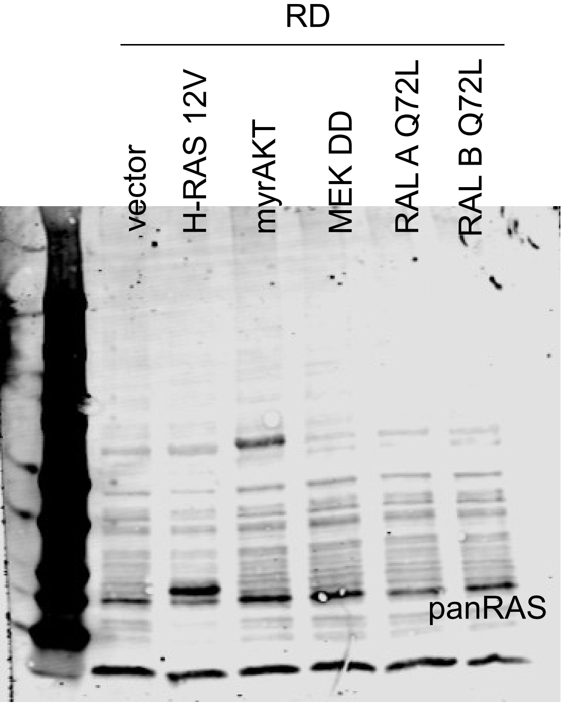

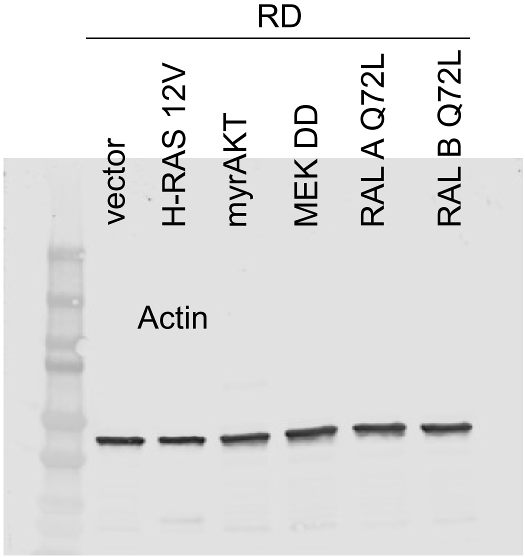

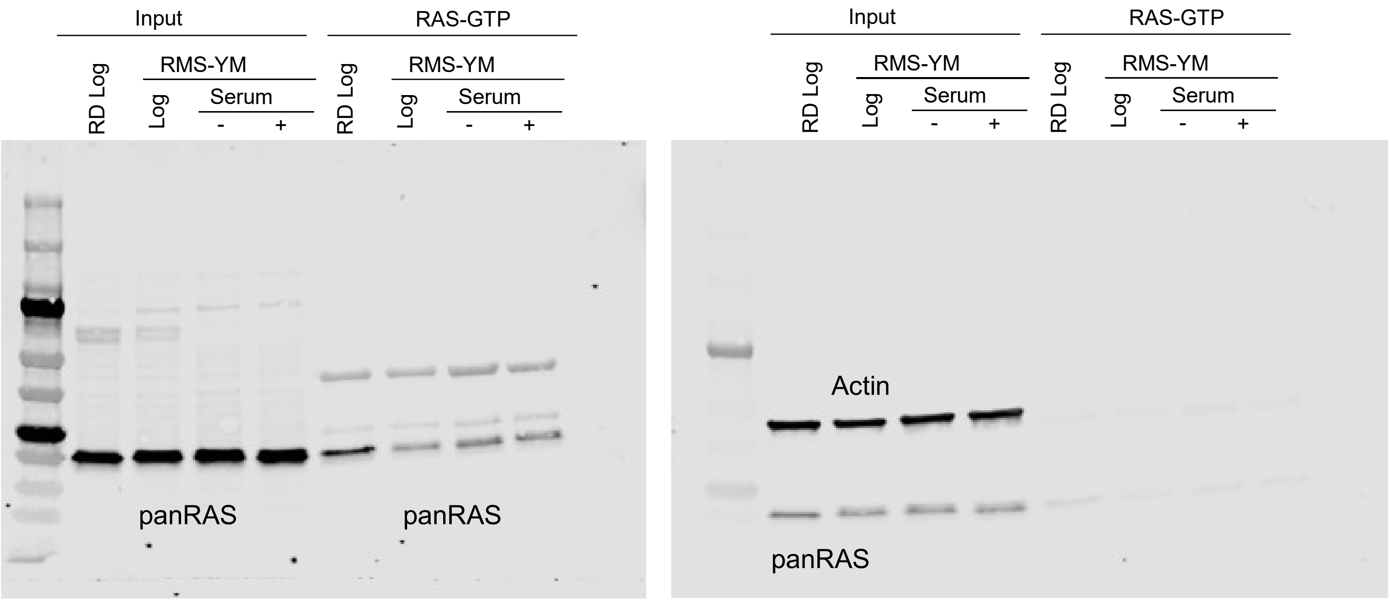

Supplementary Figure 9. Uncropped images of immunoblots corresponding to assembled data presented in Fig.6A. Included here are pan RAS corresponding to both input and RAS-GTP pull down samples and Actin.

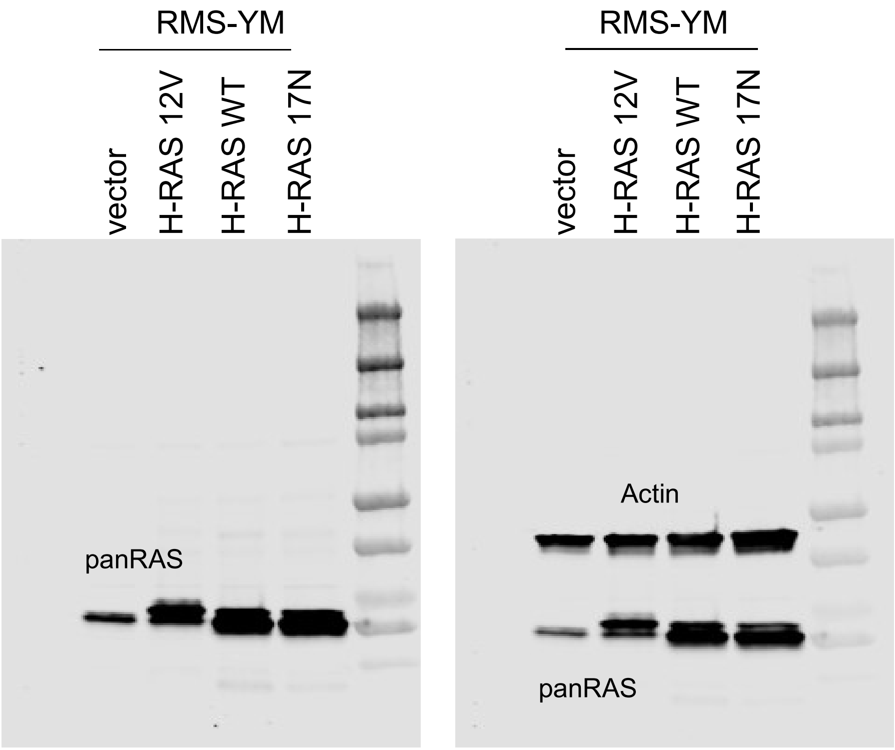

Supplementary Figure 10. Uncropped images of immunoblots corresponding to assembled date presented in Fig. 6B. Included here are panRAS and Actin

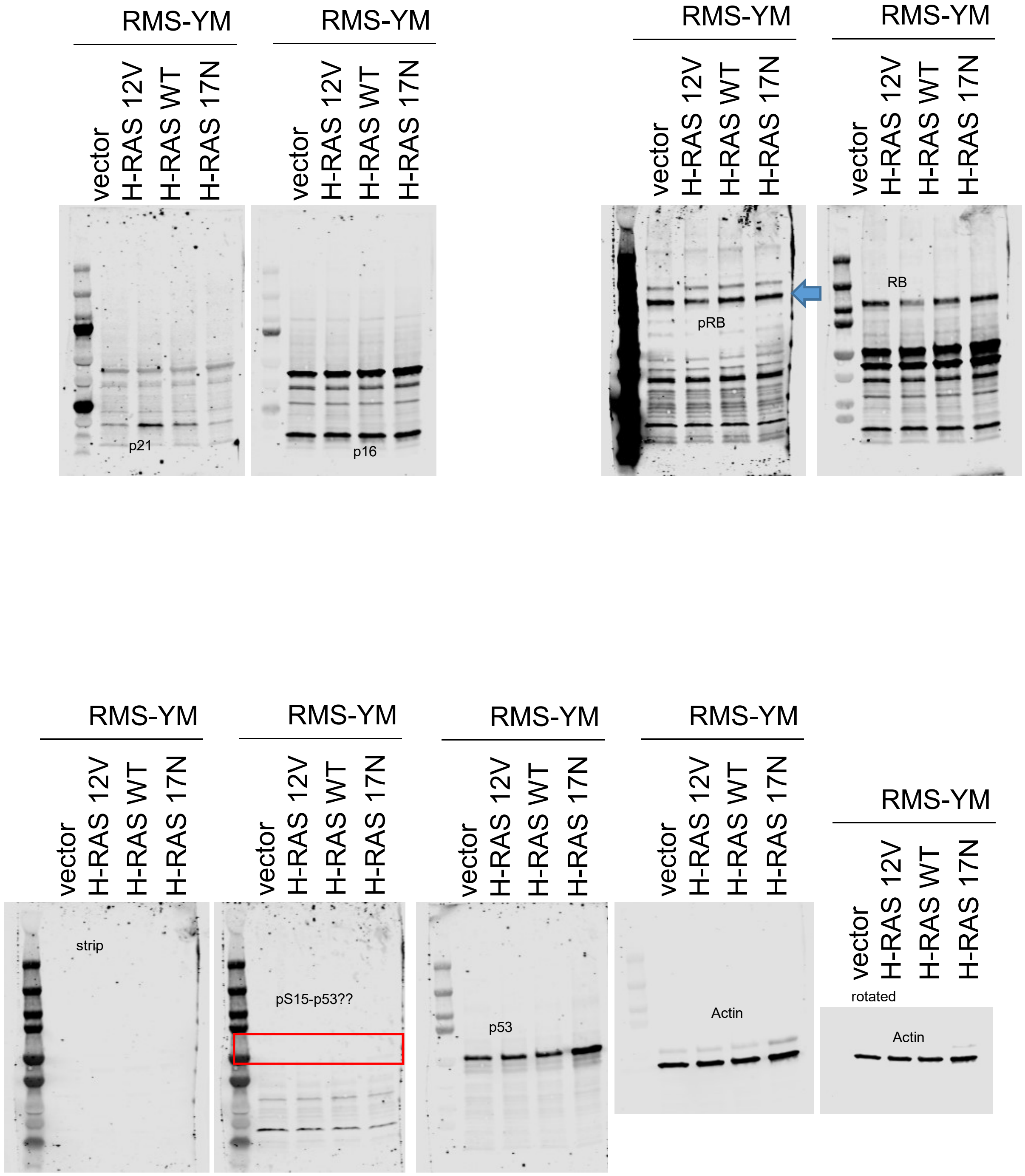

Supplementary Figure 11. Uncropped images of immunoblots corresponding to assembled data presented in Fig.6E. Included here are p21, p16, pRB, RB p53, S15-pp53, and Actin.

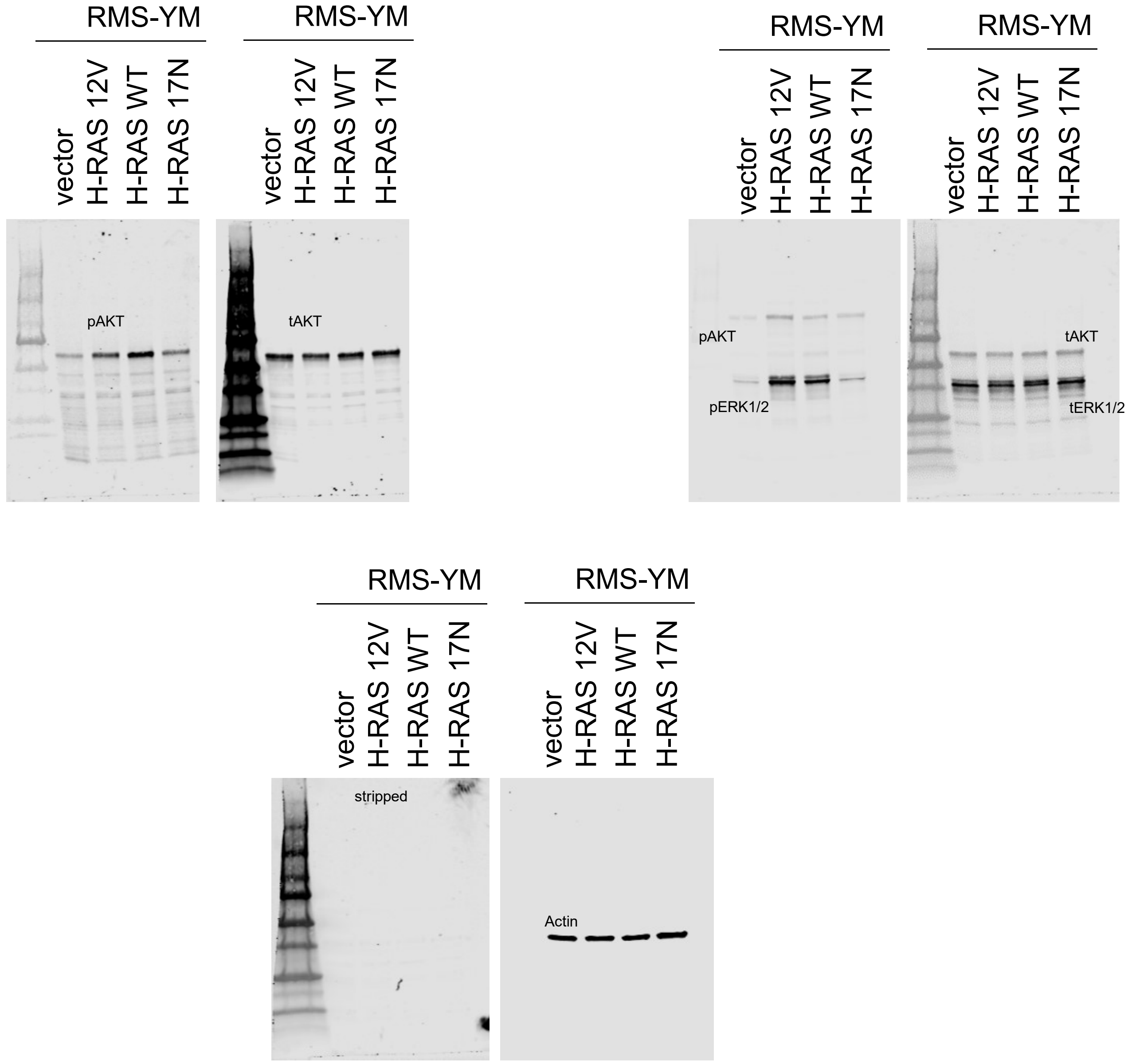

Supplementary Figure 12. Uncropped images of immunoblots corresponding to assembled data presented in Fig.6F. Included here are pAKT, total AKT, pERK1/2, total ERK1/2, and Actin.

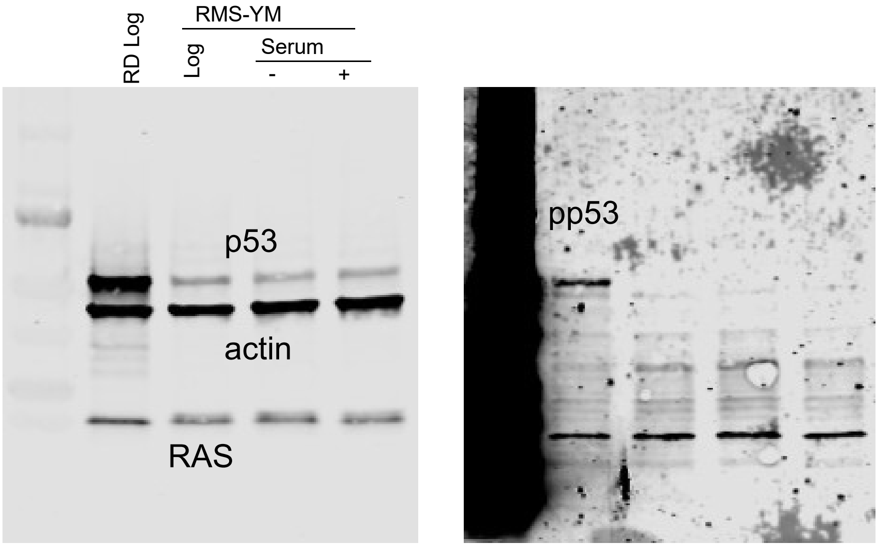

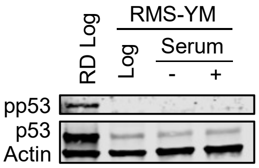

Supplementary Figure 13. Uncropped and cropped images of immunoblot showing overall p53 expression is significantly lower in RMS-YM cells compared to RD cells with Ser15 phospho p53, if present, being below limit of detection

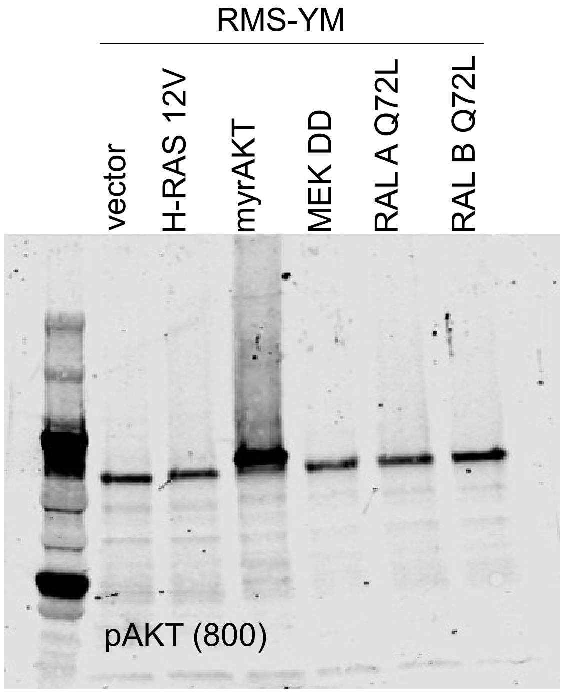

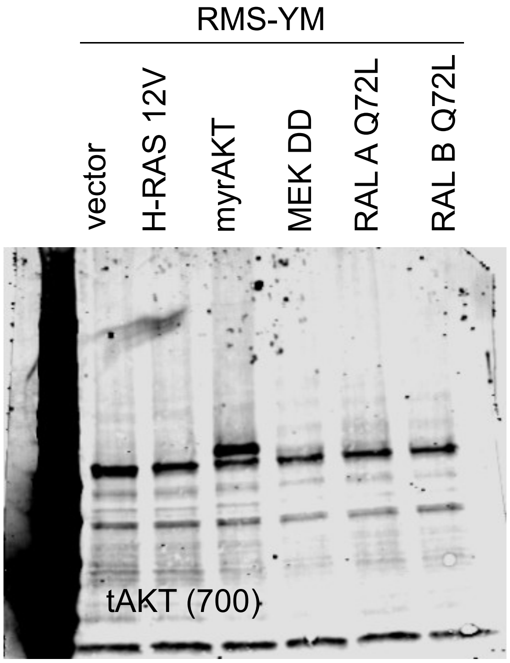

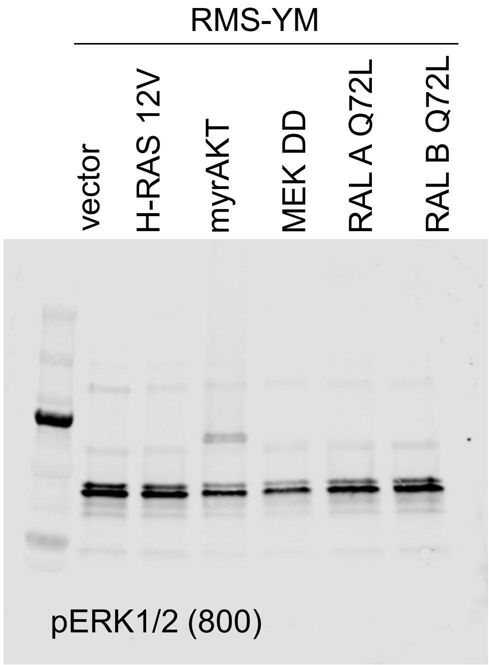

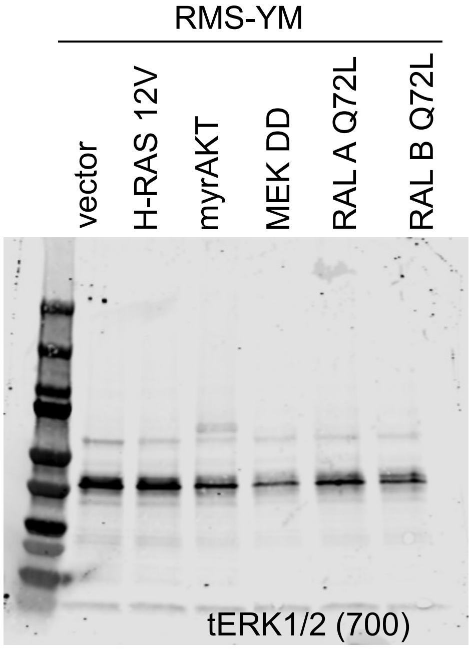

Supplementary Figure 14. Uncropped images of immunoblots corresponding to assembled data presented in Fig.8B. Included here are pAKT, total AKT, pERK1/2, total ERK1/2, pan RAS and Actin.

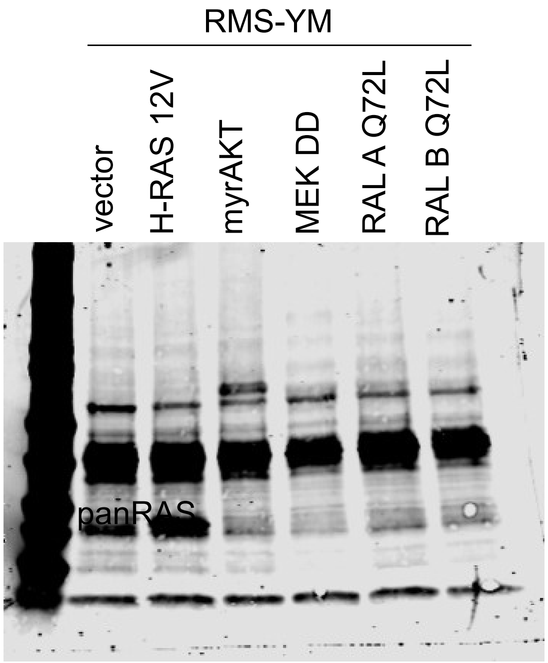

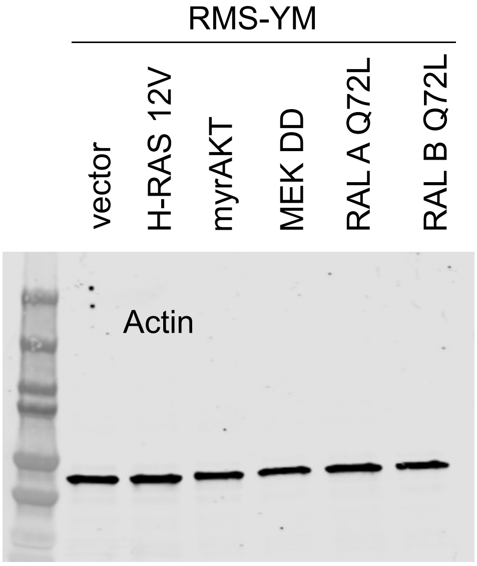

Supplement: Supplementary file 1 — Supplementary Figures. [file 41598_2021_95355_MOESM1_ESM.pdf]
